# Application of Molecularly Imprinted Polymers in the Analysis of Waters and Wastewaters

**DOI:** 10.3390/molecules26216515

**Published:** 2021-10-28

**Authors:** Mahmoud G. Metwally, Abdelaziz H. Benhawy, Reda M. Khalifa, Rasha M. El Nashar, Marek Trojanowicz

**Affiliations:** 1 Chemistry Department, Faculty of Science, Cairo University, Giza 12613, Egypt; mmetwally@sci.cu.edu.eg (M.G.M.); ahragab@sci.cu.edu.eg (A.H.B.); 201728523@std.sci.cu.edu.eg (R.M.K.); 2 Institute of Nuclear Chemistry and Technology, Dorodna 16, 03-195 Warsaw, Poland; 3 Department of Chemistry, University of Warsaw, Pasteura 1, 02-093 Warsaw, Poland

**Keywords:** molecularly imprinted polymers, solid phase extraction, heavy metals, synthetic dyes, bisphenols, personal care products, pharmaceutical compounds

## Abstract

The increase of the global population and shortage of renewable water resources urges the development of possible remedies to improve the quality and reusability of waste and contaminated water supplies. Different water pollutants, such as heavy metals, dyes, pesticides, endocrine disrupting compounds (EDCs), and pharmaceuticals, are produced through continuous technical and industrial developments that are emerging with the increasing population. Molecularly imprinted polymers (MIPs) represent a class of synthetic receptors that can be produced from different types of polymerization reactions between a target template and functional monomer(s), having functional groups specifically interacting with the template; such interactions can be tailored according to the purpose of designing the polymer and based on the nature of the target compounds. The removal of the template using suitable knocking out agents renders a recognition cavity that can specifically rebind to the target template which is the main mechanism of the applicability of MIPs in electrochemical sensors and as solid phase extraction sorbents. MIPs have unique properties in terms of stability, selectivity, and resistance to acids and bases besides being of low cost and simple to prepare; thus, they are excellent materials to be used for water analysis. The current review represents the different applications of MIPs in the past five years for the detection of different classes of water and wastewater contaminants and possible approaches for future applications.

## 1. Introduction

Water is a commodity and a sustained resource for life of all Earth’s organisms, from the simplest to the most complex. Water is used by every animal and plant, starting from photosynthesis of plants going to human’s and animals’ dependency on these plants. Thus, it is an essential element and has an important role in all biological associations, as it is used by all living organisms for specific and vital purposes [1].

Different water pollutants, such as heavy metals, dyes, pesticides, endocrine disrupting compounds (EDCs), and pharmaceuticals, are produced through continuous technical and industrial developments emerging with population increase [2]. Thus, worldwide, the major environmental concern is the treatment of water pollution, especially pollutants from wastewater, freshwater, and human and environmental pollution. Many traditional processes are applied, such as physical adsorption, oxidation, sedimentation, coagulation, and bioremediation, but they are very often not sufficiently efficient.

Most pollutants are typically concentrated in parts per billion (micrograms per liter) or parts per trillion (nanograms per liter); thus, many of the reported traditional methods are not efficient or sensitive enough for the removal and detection of pollutants. This creates a need for more efficient adsorption and preconcentration approaches. Molecularly imprinted polymers (MIPs) represent a class of promising materials that can be used in environmental sciences due to their different synthesis approaches that can be tailored according to the required target. MIPs are reported to have superior properties being easy to prepare, ecofriendly, cost-effective, and sensitive to different classes of pollutants that are expected to be found in wastewater [1,3].

## 2. Molecularly Imprinted Polymers (MIPs)

Molecularly imprinted polymers (MIPs) are synthetic materials produced from different types of polymerization reactions between a cross-linking agent with a complex formed between a template and functional monomer(s) having functional groups specifically interacting with the template through covalent or noncovalent approach. Then, when removing the template, using a highly polar solvent, a specific cavity for the target template is rendered to be ready for analysis and determination in a different matrix [3,4,5].

The history of molecularly imprinted polymers (MIPs) goes back to 1972, when Wulff et al. were able to form MIPs from organic polymers using covalent bonds, knowing that the first molecular imprinting was in 1949 when Dickey imprinted silica gels [1]. A noncovalent approach was adopted in 1981 by Arshady and Mosbach [6], which combined template and in situ monomer with noncovalent interactions, such as electrostatic forces, hydrogen bonding, van der Waals forces, etc. The weak interactions make this approach relatively simple, especially with the availability of so many functional monomers that can interact with different types of templates [3]. The development is continuous until the first MIP-based sensor was produced by Piletsky (1992), and is still under way to use polymers in different areas for various applications [7].

## 3. Synthesis of Molecularly Imprinted Polymers

**Figure 1:**
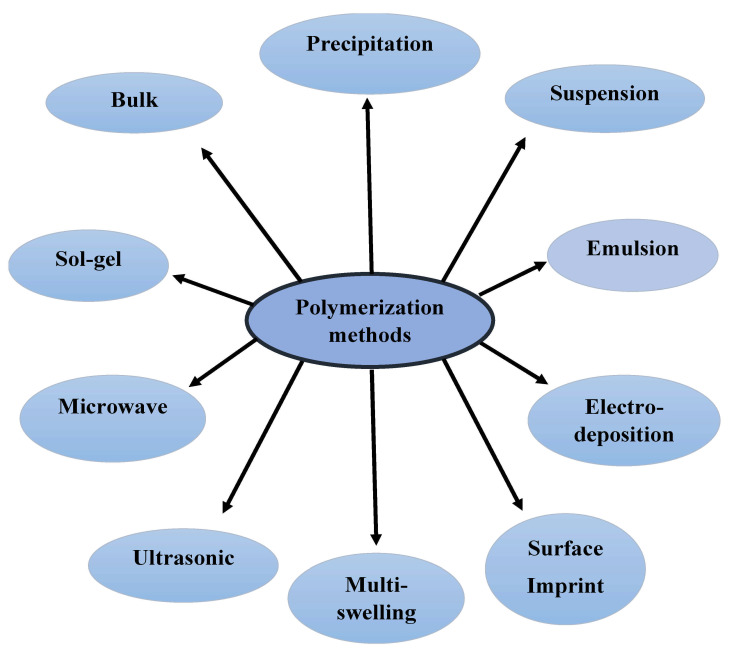
The most common polymerization methods used for MIP synthesis.

MIPs are selective sorbents to extract a target molecule in a similar approach of a “lock-and-key” or antibody-to-antigen mechanism found in the natural biorecognition. The MIPs synthesis consists of a template, monomer(s), and cross-linking agent in presence of a porogen (solvent) responsible for the distribution of the specific binding cavities “described in other simpler word as pores [8]”. The selection of the polymerization method is very important factor, as it depends on size and formats of desired MIPs besides its nature and thermal stability. Today, there are many methods for the polymerization of MIPs, as summarized in Figure 1.

### 3.1. Bulk Polymerization

This method is the most commonly used approach, as it has shown to be very easy, simple, and effective; the produced polymer can be grained and sieved according to the particle size formats desired in the target application. This method involves first the formation of a pre-polymerization complex between the template and functional monomer with suitable functional groups to form non-covalent interactions followed by the addition of a cross-linker and the initiator in a suitable solvent with certain ratios that can be optimized according to the resulting rebinding properties of the synthesized polymer. Using an insufficient amount of a crosslinker decreases the structural stability of the polymer, leading to the shedding of functional monomers, while an excessive amount of cross-linking agent reduces the number of recognition sites of MIP, and reduces its binding efficiency. After deoxygenating, degassing, and applying thermal or photoinitiation, the polymer is formed. The resulting polymer can then be ground and sieved to obtain the desired average particles size according to the concerned application.

The main drawback of such approach is the irregularity in shape of resulting particles due to the grinding step that may reduce the recognition capacity and affects the applicability of polymers produced via such a polymerization reaction in chromatographic stationary phases, even though there are many reported applications; however, it has shown to be very efficient as modifiers for different types of electrochemical sensors [9]. The wasting process is time consuming and requires high amounts of washing solvents [10,11]. One of the major drawbacks is the comparably high amounts of template required for the synthesis, which is economically inefficient in case-expensive templates [12].

### 3.2. Precipitation Polymerization

Precipitation polymerization is a relatively simple one-step method for the formation of micro- or sub-micrometer polymeric beads under appropriate conditions. The main difference between this method and bulk polymerization is the large volume of polymerization solvent in case of precipitation method. Starting with a dilute homogeneous monomer solution, the growing polymeric beads precipitate out from the solution, owing to their low solubility in the solvent. Remarkably, precipitation polymerization has been mainly used for the preparation of high-quality MIP beads with no need for stabilizer or surfactant [13,14].

The main disadvantages involve the excessive use of porogen in the polymerization process and the high amounts of template required for the synthesis. Yet, it is one of the most commonly used approaches to overcome the irregularity in shape of resulting particles when using bulk polymerization drawbacks.

### 3.3. Suspension Polymerization

Suspension polymerization is one of the promising methods for the production of uniform beads because of the use of monomers that are either insoluble or partially soluble in water. Thus, the method is actually performed through the droplets of the monomer suspended in the aqueous phase [15,16].

Even though MIPs prepared by bulk and precipitation polymerization methods demonstrate high recognition property, there might be some difficulty in the diffusion of the template to the imprinted cavities through the highly cross-linked polymeric matrix, which limits their applications for chromatography. That is why suspension polymerization to overcome this drawback through grafting of MIP layers onto the surface of synthesized beads is performed, producing MIP beads with a uniform size.

Though having many advantages, such as a homogeneous transmission of heat, this method can be used for industrial scale-up; on the other hand, the resulting number of binding sites might be low due to the use of aqueous dispersing agent and water, which might retard the reaction kinetics between the functional monomer and template molecule [1].

### 3.4. Emulsion Polymerization

Emulsion polymerization is a biphasic system [17]. The technique involves emulsifying the cross-linkers, template, and functional monomers in an aqueous solution. Stabilizers are added to the non-aqueous phase to give small, stabilized, homogeneous-sized emulsion droplets. Water-soluble redox initiator is commonly used in emulsion polymerization. The initiator decomposes under the condition of illumination or heating to generate free radicals. The functional monomers and the template molecules are first pre-polymerized, and the pre-polymerization time is generally 12 h. In order to improve the pre-polymerization effect, ultrasonic assistance can be used. Then, cross-linking agents and initiators are added to carry out a polymerization reaction under suitable conditions. Finally, the obtained polymers are eluted under appropriate conditions to elute the template molecules. 

This method forms homogeneous particle sizes but has a lot of disadvantages, such as requiring complicated conditions, which makes it difficult and expensive, showing low performance of the product, and having a water phase in the system, which might have a negative effect on the performance of the resulting polymers that are supposed to be based on favorable hydrogen bonding to the template rather than water being present in the synthesis process [18,19].

### 3.5. Surface Imprinting

In this method, the template and functional monomer(s) are mixed to form pre-polymerization complexes; then, the polymerization is conducted on the surface of the solid substrate (silica, chitosan, magnetite, etc.) in the presence of initiators and cross-linking agents to form the imprinted layer. Knocking the templates out of the polymeric layer by physical or chemical methods results in the formation of three-dimensional cavities on the surface of the solid substrate [13].

This method has many advantages, i.e., being easy to prepare, widely applicable, size- and shape-controlled, offering large surface area with highly selective binding sites [20], and being reproductive and sensitive compared to other methods [21]. It enhances the mass transfer, resulting in complete removal of the template as well as providing a good accessibility to target molecules. This technique is suitable for imprinting macromolecules, including proteins, cells, and viruses, as diffusion limitation was the major issue encountered in traditional imprinting attempts. It is commonly performed on support beads, which are mainly silica beads and are also used for the deposition of imprinted polymers. This approach imparts core shell structure to the final imprinted polymer, in which, briefly, the template and monomer are directly polymerized on the support beads, thus creating an MIP shell on the core support material. Other support materials could be used based on the application, including polystyrene and chitosan.

An important class of surface imprinting involves the use of magnetite as a solid substrate. Magnetic nanoparticles (MNPs) with superparamagnetism can serve as adsorbents in the pretreatment process that offer a significantly higher surface area-to-volume ratio, resulting in much greater extraction capacity, and can be recovered efficiently with an external magnetic field. The adsorbents and substrates can be rapidly separated, free of a large amount of organic solvent, special equipment, and a slow and time-consuming processes, which are the shortcomings of traditional pretreatment methods.

This method has few limitations, including the possibility of MIP film detachment upon repeated use and difficulty in controlling the thickness of the film.

### 3.6. Electropolymerization

In situ electro-polymerization is a special class of surface imprinting on the surface of a working electrode (glassy carbon, gold, platinum, etc.) rather than a solid non-conductive support. Electropolymerization of MIPs was utilized in the late 1990s as an alternative method to the traditionally introduced free radical polymerization. This method depends on the generation of free radical species via monomers oxidation on the sensor surface due to the applied potential. After that, these radical species are adsorbed strongly on the sensor surface and subsequently form a polymeric network. By adjusting the potential and current, thin polymeric film of controlled thickness and density can be deposited in a simple way. Additionally, this method is recommended for the direct patterning and deposition of thin films on a transducer surface with high accessibility and mass transfer.

The development of MIPs synthesis based on the electrochemical method can be considered as a parallel progress in the field of MIPs, since traditionally utilized vinyl and acrylic monomers are not electropolymerizable, and accordingly, they must be replaced by other electroactive monomers. The resulting polymeric films can be classified as conducting films and non-conducting films. The main advantage of such an approach is controlling the thickness of the polymeric layer through controlling various deposition parameters, such as current density, applied voltage, and number of deposition cycles, which gives more uniform coating of the imprinted polymer onto the electrode surface [22,23]. Alternatively, the imprinted polymers can be immobilized on the surface of the electrode by either spin coating or electrospraying of the monomer/template mixture onto the surface. This approach is very applicable especially for highly expensive templates, where a very small amount is used in the imprinting stage; additionally, it is extremely fast when compared to other chemically assisted polymerization processes [18].

### 3.7. Sol-Gel Polymerization

Sol-gel synthesis occurs by dissolving a metal oxide precursor (M(OR)n) in a low molecular weight solvent medium using a catalyst (acid, base, or ions such as F^−^) followed by a hydrolysis and polycondensation step [24]. Sol-gel is a simple, manageable, and cost-effective method allows production of different types of nanomaterials or modification of polymeric surfaces. Sol-gel polymers involving the use of silica-based materials can produce imprinted selective cavities with a longer lifetime with strong and stable structures besides the high porosity and capacious nature of silica. The method is environmentally friendly, as water or ethanol are used as solvents, and performed at room temperature [25].

Other methods of polymerization may involve ultrasonic and microwave radiation. The advantages of these methods include rapid polymerization process, controlled polymer size, simple process conditions, and a high yield, but they are still of limited application compared to other, previously mentioned methods [26,27].

## 4. Applications of Molecularly Imprinted Polymers (MIPs)

**Figure 2:**
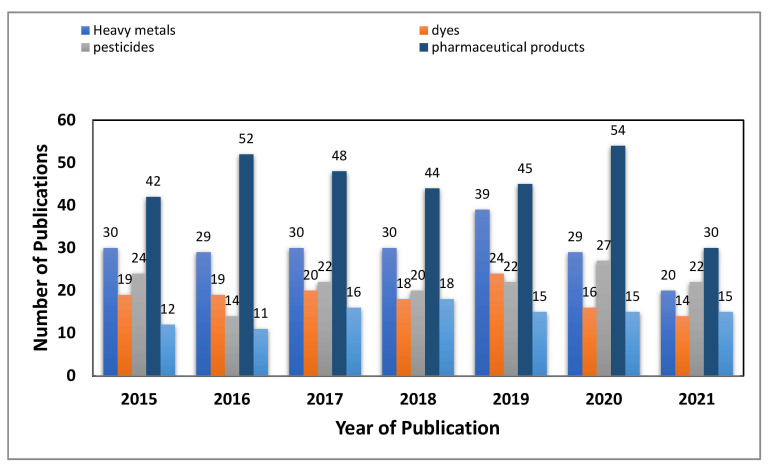
Number of molecularly imprinted polymers publications by writing “MIP” with “heavy metals”, “dyes”, “pesticides”, “pharmaceutical products” “bisphenols”, “phthalates”, and “personal care Products”, using PubMed on 15 September 2021 [29].

Nowadays, molecularly imprinted polymers (MIPs) have found vast applications in different areas, including electrochemical sensors, catalysis, immunoassays, drug delivery, solid phase extraction, and as stationary phases in selective separation applications involving chromatographic methods [28]. Figure 2 represents the frequency in the different types of MIP applications for wastewater contaminants in the past 5 years [29].

### 4.1. Molecularly Imprinted Polymers-Based Sensors

In recent years, substantial attention has been paid to the application of MIPs in the field of chemical sensors. The MIP-based sensors are designed to be used for the detection of some pollutants, such as pesticides [30], pathogens, explosives, heavy metals [31], dyes, purification of chemical and biological reagent substances [32], industrial materials, and environmental pollutants analysis [33]. MIP-based sensors for different fields, such as clinical, bioanalytical [34,35], process control, and environmental applications, represent other possible promising applications.

A device comprising a recognition element and a transducer converting the chemical’s information into a measurable signal is defined as a sensor itself. One can find even opinions that analytical techniques, such as fluid chromatography, mass spectrometry, and spectroscopic methods, will compete in the near future with such sensors. MIP-based sensors provide advantages, such as low production costs, easy storage, extended life, and the ability to be used under critical circumstances. The imprinted polymer is connected to a transducer to transform the response of MIP-based sensors into a measurable signal for such applications.

The sensitivity of the resulting sensors is directly affected by the affinity for the analyte shown by the imprinted polymer. Impressed particles should, therefore, be used in micro- or nanometer sizes with a high volume-to-surface ratio. The integration of the polymer with a transducer is, thus, a critical aspect in the preparation of an MIP-based sensor.

### 4.2. Solid-Phase Extraction with Molecularly Imprinted Polymers

The implementation of MIPs as solid phase extraction (MISPE) adsorbents has been described by numerous reports (MISPE) and the number of papers reveals an exponential increase [30,36,37]. The potential merit of MIP-based SPE lies in the ability to selectively bind to a specific target in presence of its structural analogs from a complex matrix. The application of MIP sorbents allows not only preconcentration and cleaning of the sample, but also selective extraction of the target analyte, using a very small amount of imprinted polymer (typically 5 to 200 mg) packed into a cartridge. MISPE is an efficient approach for the isolation and preconcentration of analytes from complex matrices and was successfully reported for environmental waters [38], soils [39], sediments [40], plant extracts [41], and soy [42].

The current review aims at tracking the fundamentals of molecularly imprinted polymers and their recent applications for the sensing, extraction, and determination of major water pollutant, including heavy metals, dyes, pesticides, endocrine disrupting compounds (EDCs), and pharmaceutical residues in the past five years.

## 5. Application of MIPs for the Detection of Different Classes of Contaminants

Based on literature, many methods are given in literature involving the detection and extraction of molecularly imprinted polymers as Shown in Table 1 and Table 2. Some of these applications will be discussed hereunder.

### 5.1. Heavy Metals

Water pollution is caused by various types of chemical contaminants resulting from different industries and agricultural applications, one of the most dangerous of which are heavy metals. Among many different chemically and physically applied processes, molecularly imprinted polymers represent an excellent candidate, being of low cost, stable, and in many cases sufficiently selective compared to other traditional extraction methods [43]. Two very recent reviews discussed the different approaches involving the use of molecularly imprinted polymers for detection of toxic heavy metals [44,45]. Table 1 and Table 2 represent examples of different approaches for the detection of some heavy metals using ion-imprinted polymers (IIPs), applying electrochemical sensors and solid phase extraction.

#### 5.1.1. Mercury

Mercury is a naturally occurring metal resulting primarily from Earth’s geothermal reactions. Mercury’s toxicity can be tested through protein precipitation, enzyme inhibition, and corrosive action. The most poisonous form of mercury is methylmercury, which has a high affinity for sulfuryl ligands in amino acids, and thus, it may cause protein structure changes and loss of function. Among all environmental heavy metal pollutants, mercury contamination is one of the most studied due to its persistent accumulation in aquatic environments [45]; thus, IIPs were tested for selective detection and extraction Hg(II) in water samples.

**Figure 3:**
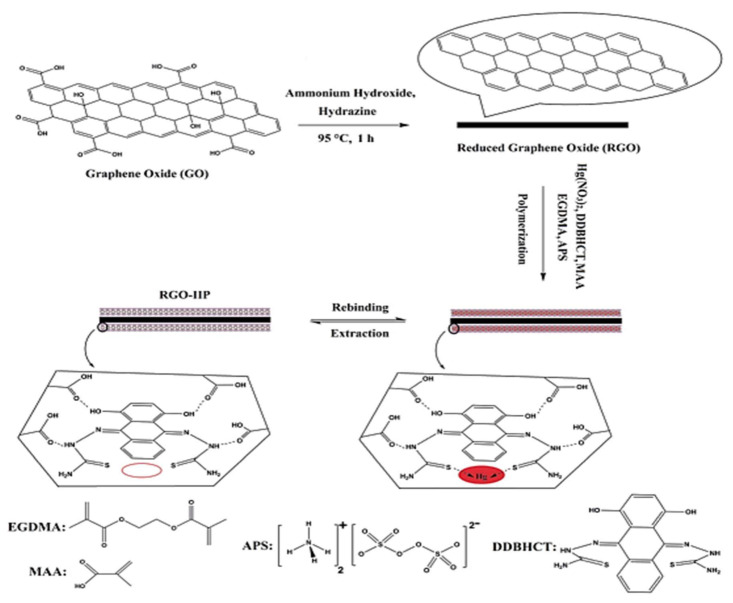
Preparation of reduced graphene oxide-ion-imprinted polymer modified glassy carbon electrode [46]. (Reproduced under permission from Elsevier, doi:10.1016/j.msec.2016.03.005).

A novel Hg(II) electrochemical sensor, based on a reduced graphene oxide (RGO) surface imprinted modified glassy carbon electrode (GCE) using methacrylic acid (MAA) as the functional monomer, ethylene glycol dimethacrylate (EGDMA) as the cross-linker, 2,2′–((9E,10E)–1,4–dihydroxyanthracene–9,10–diylidene)bis(hydrazine–1 carbothioaide) (DDBHCT) as the chelating agent, and ammonium persulfate (APS) as initiator, is shown in Figure 3. The accumulation of Hg(II) ions at the surface of a modified glassy carbon electrode was used for tracing the Hg content electrochemically within a range of 0.07 to 80 μg L^−1^. The limit of detection (LOD) was found to be 0.02 μg L^−1^ (S/N = 3), which lies below the guideline value from the World Health Organization (WHO) [46].

Another approach was developed based on sulfur containing carboxymethyl ion-imprinted polymers (S-IIPs). A bare glassy carbon electrode was modified by electropolymerizing a layer of pyrrole and then drop coating with S-IIPs. The developed ion-imprinted polymer electrochemical sensor was applied for the detection of Hg(II) in water. A detection limit of 0.1 mg L^−1^ was calculated within a concentration range of 20–800 mg L^−1^, which is lower than the WHO threshold (equal to 0.001 mgL^−1^). The results in real sample in comparison with inductively coupled plasma-optical emission spectrometry depict its potential applicability for Hg analysis [47].

#### 5.1.2. Lead

As a heavy metal element, lead is one of the most toxic to both animals and humans. It has severe toxicological effects on living organisms, and it can be transferred from soil to plants, and then to animals and humans through the food chain, resulting in severe contamination. Due to its non-biodegradability, lead accumulates through its association with inorganic and organic matter, such as by means of adsorption processes, formation of complexes, or chemical reactions [48]. Lead is a component in many industrial products, such as paints, cables, pipelines, and pesticides, and the main anthropogenic input is through the fossil fuel of combustion engines [43,45].

A solid phase extraction method based on lead ion-imprinted polymers (Pb-IIPs) using 8-hydroxyquinoline grafted gelatin and chitosan as a monomer and genipin as a cross linker. Three-dimensional IIPs were prepared with a high adsorption capacity, i.e., 235.7 mg g^−1^, and a detection limit of 0.2 ng mL^−1^. The adsorption/desorption interactions were tracked using spectrophotometric detection at 560 nm within a linear range of 1–100 ng mL^−1^ [49].

#### 5.1.3. Copper

Copper is found to be harmful to the environment and organisms in large quantities. It is well known that copper pollution has a negative impact on urban ecosystems, as well as affecting aquatic animals’ chemoreception and chemosensory [43]. WHO indicated 2 mg L^−1^ as acceptable level due to its hazardous effect on human health, leading to hypochromic anemia, osteoporosis, liver, and kidney failures and immunotoxicity [45,50].

A novel copper (II)-selective potentiometric sensor based on graphite oxide/imprinted polymer composite was developed. 5-methyl-2-thiozylmethacrylamide was used as a functional monomer and the produced Cu IIP/multiwalled carbon nanotubes/graphene oxide was used as a modifier for a carbon paste electrode. A detection limit of 4 × 10^−7^ M was reported with a linear response range 1 × 10^−6^–1 × 10^−1^ M. The electrode was applied in the analysis of different water samples (tap, river, dam) and they were in good agreement with those obtained by using inductively coupled plasma–mass spectrometry (ICP-MS) [51].

**Figure 4:**
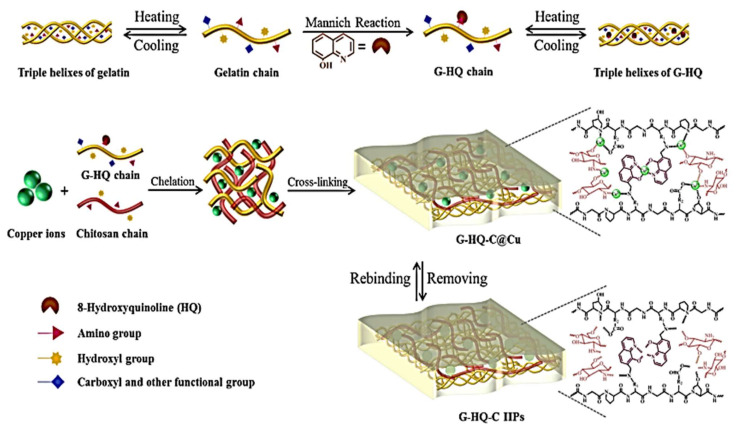
Schematic illustration of the preparation process for gelatin-hydroxyquinoline-Chitosan/ion-imprinted polymers for copper ions [52]. (Reproduced under permission from Elsevier, doi:10.1016/j.jcis.2019.01.081).

Chitosan (C), gelatin (G), and 8-hydroxyquinoline (HQ) were used as a monomer to prepare green imprinted IIPs for Cu(II). Multi-point interactions from complementary functional monomers, as shown in Figure 4, indicate that the optimal mass ratio for G:HQ was 2:1, and that the optimal G-HQ:C: Cu(II) ratio was 150:150:5. The maximum adsorption capacities of G-HQ-C IIPs toward Cu(II) reached up to 111.81 mg g^−1^ at room temperature and pH 6. G-HQ-CIIPS are applied to selective Cu(II) in different water samples [52].

#### 5.1.4. Chromium

Chromium is a common heavy metal used in many industrial applications, such as leather tanning, electroplating, metal finishing, chromate preparation, dye manufacturing, paint, paper, and aluminum manufacturing. Chromium is present in the environment in two oxidation states, hexavalent Cr(VI) and trivalent Cr(III). Cr(VI) is more toxic than Cr(III) and can lead to digestive and lung cancer.

**Figure 5:**
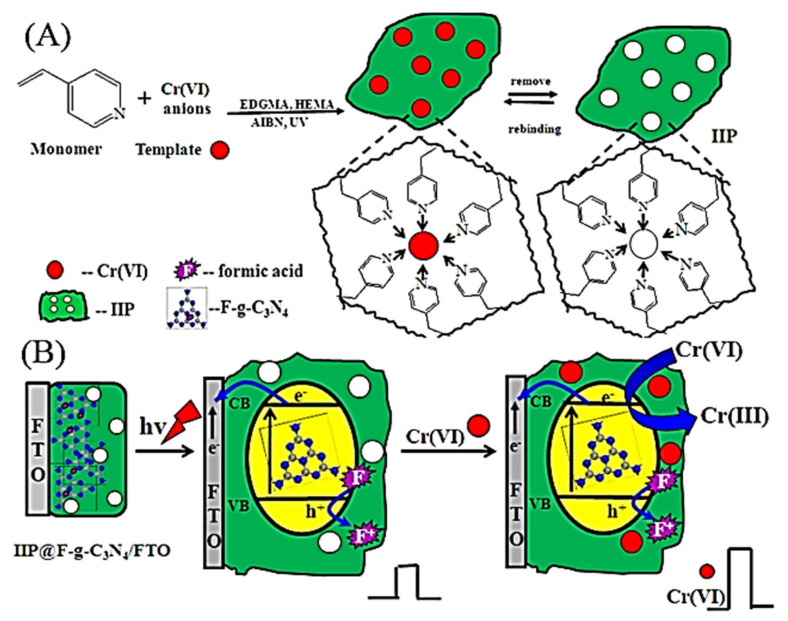
Schematic illustration of the formation process of (**A**) the IIP and (**B**) the principle of PEC determination of Cr (VI) using IIP@ graphitic carbon nitride (F-g-C_3_N_4_) [53]. (Reproduced under permission from Elsevier, doi:10.1016/j.jhazmat.2016.03.046).

A photoelectrochemical (PEC) sensor based on nano-structured hybrid formate anion incorporated in graphitic carbon nitride (F-g-C_3_N_4_) is smartly integrated with a Cr(VI) ion-imprinted polymer (IIP) (IIP@F-g-C_3_N_4_) was applied for the determination of Cr(VI) in water samples, as shown in Figure 5. The linear response to Cr(VI) concentrations in the range of 0.01 to 100 μg L^−1^ was observed with a correlation coefficient of 0.9998, and a detection limit of 0.006 μg L^−1^ (6 ppt). It is lower than those of Flame Atomic Absorption Spectrometry (FAAS) (0.8 ppb) and electrocatalytic detection (5.2 ppb). The method was used to detect Cr(VI), Cr(III), and the total chromium level in aqueous solution through the oxidation of Cr(III) to Cr(VI) and the determination of the total chromium as Cr(VI) [53].

In another study, a fluorescence sensor was developed incorporating ion-imprinted polymer (IIP) as recognition element and Mn-doped ZnS quantum dots (QDs) as fluorophores to determine Cr(VI) within a linear range of 20 μg L^−1^ to 1 mg L^−1^; the detection limit was 5.48 μg L^−1^. The prepared QDs-IIP sensor was highly specific for Cr(VI) with respect to Cr(III), Cl^−^, SO_4_^2−^, PO_4_^3−^, and MoO_4_^2−^ [54].

#### 5.1.5. Nickel

In nature, nickel is one of the most abundant metals, and has found many applications in industry, such as electroplating, batteries, and electronics [45]. Nickel is also one of the micronutrients essential for increasing hormonal activity and posturing lipid metabolism. On the other hand, excessive exposure to high levels of nickel may result in genotoxicity, haematotoxicity and carcinogenicity.

A magnetic imprinted polymer was obtained based on precipitation polymerization of amine functionalized silica-capped iron oxide particles and 4-vinyl pyridine as a complexing agent, while methacrylic acid was the functional monomer. Inductively coupled plasma-optical emission spectrometry (ICP-OES) was used for the detection during all adsorption experiments and the polymer was found to be highly sensitive for Ni^2+^ with pseudo-second-order kinetics and Langmuir isotherm. An adsorption capacity of 158.73 mg g^−1^ was obtained with limit of detection, quantification, and the percent relative standard deviation of 0.58, 1.93, and 3.4%, respectively [55].

Another nickel ion-imprinted polymers (IIPs) based on multi-walled carbon nanotubes (MWCNTs) were synthesized inverse emulsion system, using chitosan and acrylic acid as the functional monomers, Ni (II) as the template, and *N*′*N*-methylene bis-acrylamide as the cross-linker. The polymer was used as modifier for carbon paste response and cyclic voltammetry was successfully applied showing cathodic and anodic peaks at 0.018 mA and −0.016 mA, while no peaks were shown for the bare. The chemisorptions process was in line with the pseudo-second-order adsorption kinetic model and Langmuir adsorption thermodynamic model [56].

#### 5.1.6. Manganese

Manganese has found many applications in ferrous metallurgy, electrochemical and chemical processes, as well as in many foods and pharmaceutical industries. Despite being essential for some vital human process, its maximum allowed level in water should not exceed 0.1 mg L^−1^, as its accumulation has a negative impact on the nervous system [43].

A multiwalled carbon nanotube ion-imprinted polymer (MWCNT-IIP) was developed for manganese ions detection and extraction. The ion imprinted layer was synthesized on the surface of vinyl functionalized MWCNTs via free radical polymerizing using methacrylic acid as a functional monomer. A modified platinum electrode was then designed through immobilization of the above-mentioned polymer in nafion. Under the optimized conditions, Mn(II) ions could be sensed with a linear range from 1 to 5 ppm with an LOD of 0.0138 mM using differential pulse voltammetry [57].

#### 5.1.7. Cadmium

Cadmium represents a high threat toxic metal, which may lead to lung cancer, kidney, and bone damage as well as hemorrhoids. The WHO indicated that its drinking water limit should not exceed 10 μg L^−1^ [43].

Poly (N-isopropyl acrylamide) grafted and modified g-C_3_N_4_ magnetic Fe_3_O_4_ ion-imprinted polymer (IIP) was synthesized for selective interaction with cadmium have been successfully synthesized. The adsorption capacity for Cd^+2^ was found to be 2.86 times greater than that for Cu^+2^, Pb^+2^, and Zn^+2^. Pseudo-second-order model and Langmuir isotherm model fit the experimental data well [58].

Unlike commonly used sorbents for the removal of heavy metals, MIP sorbents were found to be of higher binding capacity, as the mechanism of interaction with the target metal ions not only involves physical adsorption, but also, the recognition cavities imprinted specifically for the target analyte enhances to a great extent the binding efficiency for different ion-imprinted polymers. Additionally, ion-imprinted polymeric beads could be used several times without reducing their adsorption capacity, which indicates the cost-effectiveness, taking into consideration that the knocking out of the analytes can be easily performed using different types of solvents without affecting the physical nature of the prepared polymers, which is one of the main advantages of applying the molecular imprinting approach.

**Table 1:** Application of MIPs for solid phase extraction of heavy metals.

Analyte	Monomer	Modifier	Analytical Technique	Sample	Linear Range	LOD	Adsorption Capacity	Ref.
Al(III)	APTES	Fe_3_O_4_@SiO_2_@IIPs	UV-Visible spectrophotometry	Tap and river water	5–50 ng mL^−1^	3.2 ng mL^−1^	---	[59]
Be(II)	APTES	Fe_3_O_4_@SiO2@IIPs	UV-Visible spectrophotometry	Tap and river water	2–40 ng mL^−1^	0.9 ng mL^−1^	---	[59]
Cd(II)	β-CD	Cd(ll)-IEIIP	FAAS	Waste water	---	---	86.7 mg g^−1^	[60]
Cd(II)	-	DMIP	UV-Visible spectrophotometry	Futala Lake	---	---	38.46 mg g ^−1^	[61]
Cr(VI)	MMA	Magnetic-Cr(VI) IIPs	UV-Visible spectrophotometry and ICP-OES	Real water	---	0.81 μg L^−1^	169.49 mg g^−1^	[62]
Cu(II)	(G), (C), and (HQ)	G-HQ-CIIPS	ICP-MS	River, lake and sea water	---	--	111.81mg g^−1^	[52]
Cu(II)	MAA	Cu-IIPs	FAAS	Mineral, ilam city, and river water	0.001–0.1 μg mL^−1^	0.003l μg mL^−1^	37.36 mg g^−1^	[63]
Hg(II)	MAA	Fe_3_O_4_@SiO_2_-MAPS NPs/IIPs	CE-ICP-MS	Tap water	1.25–20.83 pg mL^−1^	0.084 pg mL^−1^	25 mg g^−1^	[64]
Hg(II)	ATU	Fe_3_O_4_@SiO_2_@IIP	Flame atomic absorption spectrophotometry and an atomic fluorescence spectrometry	Tianyi lake and Ganjiang river	---	---	78.3 mg g^−1^	[65]
Hg(II)	MAA	Hg(II)-IIPs	ICP-OES	Wastewater	50 ng L^−1^–25 μg L^−1^	0.02 μg L^−1^	---	[66]
Hg(II)	2-VP	Hg(II)-IIPs	CV–AAS	Tap water and seawater	0.1–2 ng mL^−1^	5 × 10^4^ ng mL^−1^	24.6 mg g^−1^	[67]
Ni(II)	CTS	Ni(II)-IIPs	UV-Visible spectrophotometry	Environmental water	---	---	20 mg g^−1^	[68]
Ni(II)	MAA	Ni(II)-IIPs	UV-Visible spectrophotometry	Environmental water	---	---	86.3 mg g^−1^	[69]
Ni(II)	MAA	Ni(II)-IIPs	UV-Visible spectrophotometry	River, Waterfall, and tap water	3–20,000 μg L^−1^	0.001 μg mL^−1^	--	[70]
Ni(II)	CTS	Magnetic Ni(II)–imprinted chitosan nanocomposite	FAAS	River, lake, well, and spring water	0.5–50 μg L^−1^	0.06 μg L^−1^	---	[71]
Ni(II)	MAA	Ni-IIPs	FAAS	Mineral, Ilam city, and river water	0.009–1.8 μg mL^−1^	0.002 μg mL^−1^	---	[72]
Pb(II)	CMSHA	IIP-MMT	FAAS	Wastewater	200–1000 mg L^−1^	---	201.84 mg g^−1^	[73]
Pb(II)	MAA and 4-VP	H-MIPs	ICP-AES	River, tab, well, and mineral water	0–300 mg L^−1^	---	40.52 mg g^−1^	[74]
Pb(II)	2-VP	Pb(II)-IIPs	FAAS	Tap, distilled, and seawater	3–150 μg L^−1^	0.75 μg L^−1^	85.6 mg g^−1^	[48]
Pb(II)	4-VP	IIPs-SPE	FAAS	Tap, river, and seawater	3–600 μg L^–1^	0.9 μg L^−1^	9.8 mg g^−1^	[75]

**(AAS)** atomic absorption spectroscopy, **(AFS)** atomic fluorescence spectrometry, **(APTES)** 3-(aminopropyl)triethoxysilane, **(ATU)** Allylthiourea, **(BSOMe)** (4-[(E)-2-(4′-methyl-2,2′- bipyridin-4-yl)vinyl]phenyl methacrylate, **(β-CD)** β-cyclodextrin, **(CE-ICT-MS)** capillary electrophoresis-inductively coupled plasma mass spectrometry, **(CMSHA)** 5-chloromethyl-salicylhydroxamic acid, **(CTS)** Chitosan, **(DMIP)** dual molecularly imprinted polymer, **(FAAS)** flame atomic absorption spectrometry, **(G)** Gelatin, **(G-HQ-CIIPS)**, gelatin hydroxyquinoline chitosan ion-imprinted polymers, **(HEMA)** Hydroxyethylmethacrylate, **(H-MIPs)** hollow mesoporous molecularly imprinted polymers, **(HQ)** hydroxyquinoline, **(ICP-AES)** inductive coupled plasma atomic emission spectrometer, **(ICP-OES)** inductively coupled plasma optical emission spectrometer, **(IEIIP)** inverse emulsion ion-imprinted polymer, **(IIP-MMT)** montmorillonite based surface ion-imprinted polymer, **(IIPs-SPE)** ion-imprinted polymers-solid phase extraction, **(IPTS)** 3-isocyanatopropyl triethoxysilane, **(MAA)** methacrylic acid, **(MMA)** methyl methacrylate, **(Ni-MIIPs)** Nickel magnetic ion-imprinted polymers, **(TGA)** thermogravimetric analysis, **(4-VP)** 4-vinylpyridin.

**Table 2:** Determinations of heavy metal ions using voltammetric sensors modified with MIPs.

Analyte	Monomer	Modifier	Working Electrode	Analytical Technique	Sample	Linear Range	LOD	Ref.
Cd(II)	MAA	Au nanoparticles Cd(II)-IIPs	CPE	CV, DPV, EIS	Tap and river water	10^−9^–10^−4^ mol L^−1^	43 × 10^−10^ mol L^−1^	[76]
Cr(VI)	CTS	(IIP-S)	Au-plate	CV and EIS	Tap and river water	1 × 10^−9^–1 × 10^5^ mol L^−1^	6.4 × 10^−10^ mol L^−1^	[77]
Cr(VI)	4-VP	(IIP@F-g-C_3_N_4_)	FTO electrode	EIS	Tap, east lake, and river water	0.01–100 ppb	0.006 ppb	[53]
Cr(III)	MAA	MWCNT-IIP	Pt Electrode	CV, DPV	Industrial wastewater	1–5 ppm	0.051 μmol L^−1^	[78]
Cu(II)	5-methyl-2-thiozylmethacrylamide	Graphite oxide-IIPs	CPE	Multichannel potentiometric measurement system	Tap, dam, and river water	1 × 10^−6^–10 M	4 × 10^−7^ M	[51]
Hg(II)	CMC	GCE/PPY/S-IIP	GCE	SWASV/EIS	Tap, ground, and wastewater	20–800 μg L^−1^	0.1 μg L^−1^	[47]
Hg(II)	MAA	GCE-RGO-IIP	GCE	SWASV	Tap, aqueduct, river, and wastewater	0.07–80 μg L^−1^	0.02 μg L^−1^	[46]
Mn(II)	(MAA), and (NNMBA)	MWCNT-IIPs	Pt Electrode	CV, DPV	Real water	1–5 ppm	0.0138 μM	[57]
Mn(II)	MAA	Mn(II)-IIP/MWCNT/chit/IL	GCE	SWASV	Wastewater	2–9 μM	0.015 μM	[79]
Ni(II)	CTS and acrylic acid	MWCNTs-IIPs	CPE	CV	Industrial wastewater	10–40 mg L^−1^		[56]

**(AAPTS)** 3-(2-amino ethyl amino) propyl trimethoxysilane, **(CMC)** Sodium carboxymethyl cellulose, **(CPE)** carbon paste electrode **(CTS)** chitosan, **(CV)** Cyclic voltammetry, **(DMSO)** dimethyl sulfoxide, **(DPV)** differential pulse voltammeter, **(EIS)** electrochemical impedance spectroscopy, **(GCE-RGO-IIP)** glassy carbon electrode reduced graphene oxide ion-imprinted polymer, **(IIP-S)** ion-imprinted chitosan-graphene nanocomposites, **(MAA)** Methacrylic acid, **(MWCNTs-IIP)** Multi-walled carbon nanotubes, **(NNMBA)** *N*,*N*, Methylene-bis-acrylamide, **(QDs)** quantum dots, **(SWASV)** square wave anodic stripping voltammetry, **(4-VP)** 4-Vinylpyridine.

### 5.2. Dyes

Synthetic dyes are employed in many industries, such as textile, paper, plastics, leather, paints, etc. Their discharge to the environment has a negative impact, as some of these dyes are highly toxic, carcinogenic, and mutagenic in nature and are capable to bio-accumulate in the food chain, besides being stable and non-degradable. Even a very small amount of dye in water (about 10–20 mg L^−1^) is highly visible, which affects water’s transparency and gas solubility and can prevent the penetration of light and oxygen, consequently reducing the photosynthetic activity in aquatic environments. The application of molecularly imprinted polymers seems to be a very promising approach for the analysis of water samples contaminated with different types of dyes.

Congo Red (CR) is a disazo dye and metabolizes to the carcinogen benzidine. It also has a sulphonic acid group that enhances its hydrophilicity and has high stability and low biodegradability. Dispersion polymerization was first applied for polystyrene microspheres using CR as the template molecule followed by a single-step swelling polymerization to prepare CR-MIP microspheres in the aqueous phase using methacrylic acid as the functional monomer. The absorption capacity was found to be 27.0 mg g^−1^, which is comparable to other reported adsorbents. The MIP-based method is superior in terms of the stability of the reusability of the prepared polymers. Additionally, the average removal rate of CR was found to be 95.63% and 91.73% from river and wastewater, respectively, indicating the high applicability of the prepared polymers [80].

Acid Green 16 (AG16) belongs to the triphenylmethane dyes and is widely used for nylon, wool, cotton, and silk pigmentation. It has three aryl radicals bound to a central carbon atom and was found to have genotoxic and mutagenic effects. Acid Green 16 bulk polymerized imprinted polymer was developed using 1-vinylimidazole as a functional monomer and ethylene glycol dimethacrylate as the cross-linker. The polymer showed excellent rebinding to the template of 83% as indicated by the HPLC measurements with an imprinting factor of 6.91. Additionally, it showed high extraction efficiency from water samples, with almost 100% recovery compared to commercial SPE cartridges [81].

Dye Acid Violet 19 is another example of a triphenylmethane dye. A computational simulation approach was applied, upon which 1-vinyl imidazole was also selected as a functional monomer. The dye adsorption was found to be fitting with the Langmuir model, with an adsorption capacity of 6.93 mg g^−1^ compared to 2.84 mg g-1 for the corresponding non-imprinted polymer (NIP) with pseudo-second-order kinetics (k^2^ = 0.2416 mg g^−1^ min^−1^ for the MIP) and an imprinting factor of 2.89. The polymer was successfully applied for extraction, and the determination of Acid Violet 19 presents in complex real samples, with recoveries values ranging between 85 up to 99% [82].

Auramine O is among the illegally used dyes as additives in a variety of food products due to its low price, despite its reported toxicity and causing dermatitis and upper respiratory irritation symptoms. Magnetic molecularly imprinted polymer was designed for the detection of Auramine O using itaconic acid as a monomer surface imprinted on magnetite modified silica. The high specificity of the stoichiometric imprinted polymers was proven in the extraction of mixture solution of Auramine O, auramine O hydrochloride, and chrysoidine. Recoveries from lake water ranged between 99.66 and 108.75% (RSD 2.6–3.7%, *n* = 3), indicating the possibility of application of the magnetic polymers for real samples [83].

Methyl Green is a di-cationic triphenylmethane dye used for staining pharmaceutical syrups and many biological applications. Magnetic molecularly imprinted polymer based on acrylamide surface imprinted on magnetic silica was prepared. The adsorption studies revealed a pseudo-first order kinetics with maximum adsorption (Q) of 3.13 mg g^−1^ compared to 1.58 mg g^−1^ for the corresponding non-molecularly imprinted polymer (NIP). The polymer was used as a modifier for carbon paste electrode, and square-wave adsorptive anodic stripping voltammetry (SWAdASV) was applied for electrochemical analysis. A linear concentration range of methyl green from 9.9 × 10^−8^ to 1.8 × 10^−6^ M, with a limit of detection (LOD) of 1.0 × 10^−8^ M was shown. The sensor was applied for two spiked river water samples with recoveries of 93 to 103% [84].

### 5.3. Pesticides

The use of pesticides all over the world for protection against different plants hazardous infections with insects, viruses, invasive plants, fungus, and weeds has drastically increased in the past decades. Despite their protective effects, they are seriously accumulated in soils and can be transmitted to the surface water [85]. Thus, their presence and allowed WHO levels should be monitored, especially the use of forbidden types of reagents due to their toxic activity. Their hazards are not only limited to target organisms; yet, due to their chemical properties and persistence, they can negatively affect the ecosystem [86].

Many of the compounds belonging to such class are either UV- or electro-inactive, which limits the number of applications using traditional HPLC-UV detection methods and restricts the solid phase extraction techniques to Gas Chromatography (GC)- or mass detection (MS)-based chromatographic methods. On the other hand, electrochemical detection, where an electroactive probe, such as ferrocyanide, can be used when inactive analytes are used, which were widely applied in the past years using molecularly imprinted polymers. The active probe can be used to detect the interaction between the target template and its specific binding sites in the modified sensors, which affects the electrochemical signal of the probe. Thus, MIPs have found many applications for the detection of herbicides, insecticides, and fungicides, representing the most-often used classes of pesticides, as shown in Table 3.

#### 5.3.1. Insecticides

Organophosphates, a very widely used class of insecticides, can accumulate for days or weeks in the aquatic environment, affecting the ecosystem and disturbing marine life [86]. Very recently, in August 2021, the U.S. Environmental Protection Agency (EPA) has completely prohibited the use of chlorpyrifos.

MIP UV-cured solid-phase extraction discs (MISPE) were developed for chlorpyrifos detection in water samples. Two monomers, namely glyoxal bis(-diallyl acetal) and pentaerythritol tetrakis(3-mercaptopropionate), were crosslinked with polyethylene glycol diacrylate (PEGDA) and photoinitiated [116]. Gas chromatography with mass detection (GC–MS) was applied at a linear range of 0.1–7.5 mg L^−1^, with a limit of detection (LOD) and limit of quantification (LOQ) determined as 0.05, and 0.1 mg L^−1^, respectively. The MIP UV-cured discs were found to be regenerable and usable up to nine runs using 15 mL of a 9:1 methanol/acetic acid solution for 45 min followed by methanol and distilled water, and finally dried, Unfortunately, these levels are still much higher in relation to the new regulations, which urges further developments to be made so as to enhance the sensitivity of the extraction methods.

Gold microelectrodes modified using electropolymerized pyrrole (Py) were applied for the detection of chlorpyrifos (CPF). Differential pulse voltammetry (DPV) and electrochemical impedance spectroscopy (EIS) techniques were used for measurements. The voltammograms show a decrease in the peak current value from 3.9 to 0.27 μA as CPF concentrations increased from 1 fM to 1 μM. This is a consequence of CPF rebinding at the cavities in the PPy matrix, which primarily occurs via hydrogen bonding between the N group of pyridine in CPF and the N−H group of PPy. Recovery from spiked cucumber and pomegranate ranged from 91 to 97% with an RSD of 5% [117].

Another study reports the use of GCE modified with carbon nitride nanotubes (C_3_N_4_ NTs) coated with graphene quantum dots (GQDs) electrodeposited pyrrole for the detection of chlorpyrifos (CPF). The sensor’s applicability in wastewater samples showed a limit of detection of 2.0 × 10^−3^ nM, with a linear range of 1.0 × 10^−2^–1 nM using SWV for electrochemical measurements. The results for electrochemical methods are much more sensitive compared to other chromatographic methods, as indicated by the very low LOD.

Magnetic imprinted polymers were prepared and characterized for the detection of 12 organophosphorus pesticides OPPs. Methyl-parathion (MP) and/or quinalphos (QP) were chosen as templates for the synthesis of dual template or single-template MIPs. The polymer showed high affinity and good recognition for all twelve OPPs including quinalphos, isazophos, chlorpyrifos-methyl, chlorpyrifos, methidathion, triazophos, profenofos, fenthion, fenitrothion, methyl-parathion, parathion, and paraoxon. GC-MS results were attained within 10 min of interaction with the different templates, with a limit of detection of 1.62–13.9 ng g^−1^ and spiked recoveries of 81.5–113.4%. Linear calibration plots were found in the range 10–800 ng mL^−1^, indicating the applicability of the developed polymers for rapid determination and high throughput analysis of multi-pesticide residues [118].

As methyl-parathion is electroactive, MIP-based electrochemical sensors prepared using both electro- and bulk polymerization was applied for its detection [112]. Electropolymerized p-aminothiophenol was used as functional monomer on GCE modified with gold nanoparticles coated with carbon nanotubes. Linear sweep voltammetry results showed a linear range 0.38–4.2 nM and 4.2–42 nM, with a limit of detection of 0.30 nM for spiked distilled and tap water samples.

Modified carbon paste electrodes (CPE) were applied for the detection of parathion [113] and diazinon [109], where the MIPs were synthesized by the bulk polymerization method and utilizing MAA as the functional monomer. The results for parathion detection in tap, river, and lake water showed LOD 0.5 nM and linear range 1.7 × 10^2^–9.0 × 10^2^ nM by using SWV after multiwalled carbon nanotubes (MWNTs) modification. Detection of diazinon in tap and river water sample was performed successfully with LOD 0.13 nM and linear range 0.5–1.0 × 10^3^ nM.

**Figure 6:**
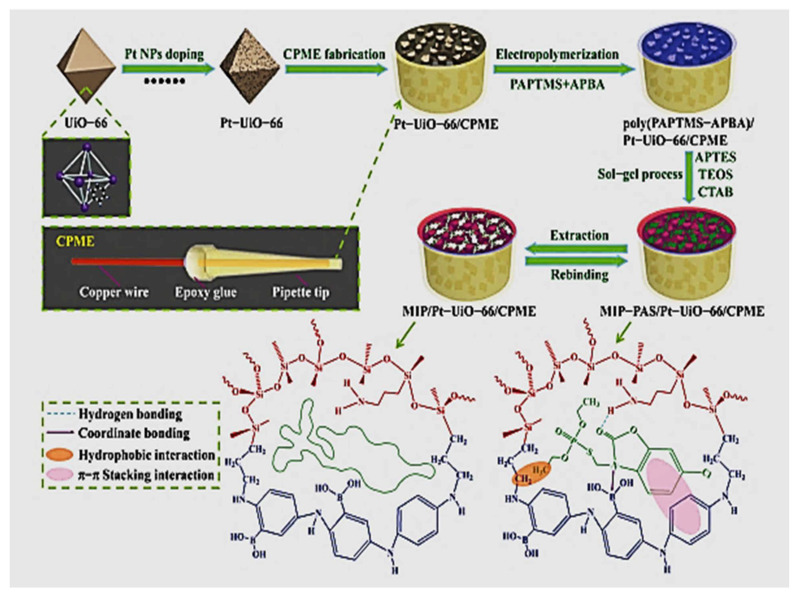
Schematic representation of the template extraction-rebinding concept and the production of MIP films on Zr-based metal–organic framework catalyst (Pt-UiO-66)/carbon paste microelectrode [119]. (RSC open access content, further permissions related to this figure should be directed to the RCS, doi:10.1039/d0an00278j).

Metal organic frameworks (MOFs) connected by self-assembly with transition metal ions/clusters and organic ligands have seen a rise in scientific interest in recent years, as they led to improving the response characteristics in the detection of some pesticides. A disposable molecularly imprinted electrochemical sensor was developed for phosalone (PAS), employing home-made carbon paste microelectrode (CPME) modified with a Zr-based metal−organic framework catalyst (Pt−UiO−66) and mesoporous structured conductive molecularly imprinted polymer. The MIP was prepared using the sol-gel method with 3-aminopropyltriethoxysilane (APTES) as the functional monomer, and UiO-66 was mixed with Pt nanoparticles as modifier as shown in Figure 6. The sensors response for lake water samples showed limit of detection 0.078 nM and linear range 0.50–2.0 × 10^4^ nM [119].

Cypermethrin (CYP) is a pyrethroid insecticide that is more effective and less harmful than organophosphates in terms of insecticidal activity. A surface-enhanced Raman spectroscopy (SERS)-active substrates were developed using Au nanoparticles (NPs) coated with a layer of polymer and followed by imprinting with a pesticide—Cypermethrin. The captured molecules situated in between the areas of high electromagnetic field formed by plasmonic Au NPs result in an effect of SERS. The results showed that Au NP/MIP was competent to detect both similar molecules despite the imprint being made only by cypermethrin. Nevertheless, Au NP/MIP has a limited number of imprinted cavities that resulted in sensing only low concentrations of a pesticide solution. Au NP/MIP is, thus, a specific design for detecting analogous molecules similar to its template structure [120]. The optical microscope of the spectrometer was set at 50 magnification and the He-Ne Raman lasers used have a diameter of 1 m; laser excitation wavelengths of 633 and 785 nm with gratings at 1800 and 1200 lines/nm, respectively, were used.

Cypermethrin in spiked wastewater samples was detected employing an MIP sensor based on the polymerization of phenol as a functional monomer on glassy carbon electrode modified with core-shell type nanoparticles (Fe@AuNPs), including two-dimensional hexagonal boron nitride (2D-hBN) nanosheets [107]. The produced nanocomposites have good sensitivity and selectivity in the detection of CYP with LOD 3.0 × 10 ^5^ nM and linear range 1.0 × 10^−3^–10 nM.

**Figure 7:**
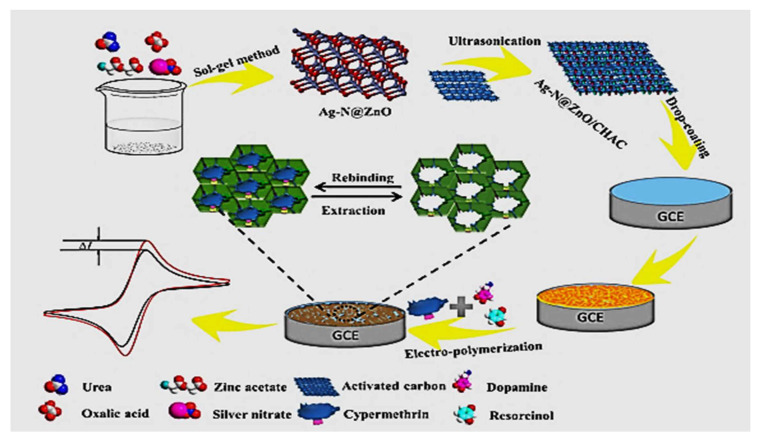
Schematic preparation method of DMMIP-Ag-N@ZnO/CHAC/GCE for the detection of cypermethrin [108]. (Reproduced under permission from Elsevier, doi:10.1016/j.bios.2018.12.002).

Another hybrid electrochemical sensor for sensitive detection of cypermethrin (CYP) was also reported. Firstly, Ag and N co-doped zinc oxide (Ag-N@ZnO) was produced by the sol-gel method, and then Ag-N@ZnO was ultrasonically supported on activated carbon prepared from coconut husk (Ag-N@ZnO/CHAC). Finally, a layer of MIP was fabricated in situ on a glassy carbon electrode by electropolymerization, with dopamine and resorcinol as dual functional monomers (DM) and CYP acting as a template (DMMIP-Ag-N@ZnO/CHAC), as shown in Figure 7. Under the optimal conditions, a calibration curve of current shift versus concentration of CYP was obtained in the range of 2 × 10^−13^~8 × 10^−9^ M, and the developed sensor gave a remarkably low detection limit (LOD) of 6.7 × 10^−14^ M (S/N = 3) [108].

The sensitivity and selectivity can be improved by combining electrochemiluminescence (ECL) with MIPs. Triazophos were detected using p-aminothiophenol (PATP) as a functional monomer and modified it with luminol on a gold electrode. The results of the detection of triazophos in tap water, reservoir water, and river water showed an LOD of 0.058 nM and a linear range of 0.1–1.0 × 10^3^ nM [115]. Quantum dots were used as the luminophore and hydrogen peroxide as a co-reactant oxidized or reduced at the electrode in the electrochemical luminescence process, resulting in the emission of radiation [121]. The result from measurement for Cyfluthrin in the seawater sample showed that there was an LOD of 0.12 nM and a linear range of 0.46–2.3 × 10^2^ nM.

#### 5.3.2. Herbicides

2,4-dichlorophenol (2,4-DCP) is a common chlorophenol used in the production of herbicides [94]. 2,4-DCP-MIP is an electrochemical sensor used for the detection of 2,4-DCP, utilizing the functional monomer (MAA) and a mixture of chitosan (Ch) and nafion to immobilize the MIP and improve conductivity. The mass transfer to the electrode surface was likely blocked by this assembly, resulting in an LOD of 1.6 × 10^3^ nM.

Carbon fiber paper can be used as the working electrode to detect 2,4-DCP using a poly(3,4-ethylenedioxythiophene) (PEDOT)-based MIP [95]. Using PEDOT as a conductive polymer, the result showed a limit of detection of 0.07 nM. In a different approach [92], GCE modified with o-PD was electropolymerized with multifunctional nanomaterials, zinc oxide nanoparticles (ZnO NPs), and graphene platelets to create the MIP. This MIP was evaluated in real wastewater samples, and exhibited higher sensitivity than other approaches used for the detection of 4-CP, with a limit of detection of 40 nM and a linear range of 2.0 × 10^2^–1.7 × 10^5^ nM.

Bensulfuron-methyl (BSM) was detected using imprinted magnetic microspheres sorbent [89], prepared by trimethylolpropane trimethacrylate (TRIM) as a cross linker and MAA as a functional monomer to coat SiO_2_ beads to get high stability. HPLC was used for detection with a linear range of 0.04−0.8 µM and a limit of detection of 6.4−9.5 nM, while the adsorption capacity was 37.32 mg g^−1^. The polymer has high reproducibility when reused up to six times.

A new method for the preparation of MIP-coated hollow fibers, using toluene, methacrylic acid (MAA), 2,2′‒azobisisobutyronitrile (AIBN), and ethylene glycol dimethacrylate (EGDMA) as solvent, functional monomer, initiator, and cross-linker, respectively, was applied for solid phase micro extraction of herbicides of propazine (PPZ) in aqueous media [100]. This method requires an incubation time of 1 h, and an adsorption capacity of 5.5 µg per molecularly imprinted polymer-coated hollow fiber (MIP-HFs). The MIP-HFs can detect propazine (PPZ) with a linear range of 0.1–25 µg L^−1^ and a limit of detection of 0.03–0.1 µg L^−1^ when using HPLC-DAD detection. Yet, the recovery is not that high and further optimization is required to improve efficiency. Another method for PPZ determination was developed with supported liquid membrane molecularly imprinted beads based on biphasic solvent toluene and acetonitrile, Di VinylBenzene (DVB) as crosslinker, AIBN as initiator, and MAA as functional monomer [101]. The optimized elution time was found to be half an hour with the elution solvent methanol, with high reproducibility on reuse up to reach to 30 times with HPLC-DAD with a limit of detection 0.022–0.030 µg L^−1^. This method uses minimum solvent and can be called eco-friendly with good recognition abilities.

Based on the above presented examples, unlike traditional methods, MIPs are promising sorbents for the detection of pesticide residues with various applications in the separation of analytes in a complex matrix coupled with chemical sensors and have been used directly and indirectly with chromatographic techniques. Different types of real samples, including fruits, vegetables, and biological materials, that are very difficult to analyze and have high matrix effects, especially on fats and proteins, which often interfere in the traditional detection approaches for pesticide. MIPs are proven to be excellent sorbents for the detection of pesticides in real samples.

Bulk polymerization using methacrylic acid (MAA) was widely applied as a functional monomer. While EGDMA and DVB are the most commonly used cross-linker for stabilizing the pre-polymerization complex formed between the monomer and the target template by fixing the functional monomer around the template molecule. The proper adjustment of the cross-linker type and ratio in the polymerization process has a great effect on the properties of the resulting polymer, where the increase in the amount of crosslinker may reduce the number of imprinted recognition sites and affect the rigidity of the polymer when rebinding to the template in any removal experiments, while inefficient crosslinking may result in low stability and poor polymerization complexes. Most of the reported methods applied free-radical thermal or photochemical polymerization initiator for various types of imprinting reactions. Magnetic surface molecularly imprinted polymer was found to be a highly efficient sorbents for pesticides, where an external magnetic field can be applied to collect the sorbents in a few seconds compared to other separation methods involving filtration or centrifugation. MIPs have showed higher specificity and selectivity for the target analytes, besides the possibility to be used multiple times.

### 5.4. Endocrine Disrupting Chemicals (EDCs)

Personal care products (PCPs) include many types of preparations found in pharmaceutical products and beauty stores, which can be purchased without a prescription. They include cosmetic products (moisturizers, perfumes, lipsticks, fingernail polish, facial or eye makeup, shampoos, colors for hair, kinds of toothpaste, and deodorant products), household products, food, or pharmaceuticals (skin protectants such as lip balms and diaper ointments, mouthwashes marketed for therapeutic purposes, antiperspirants, dandruff or acne treatments, etc.) [122]. In the everyday activity of human beings, huge quantities of PCPs are used, such as swimming, bathing, and other hygienic activities, at thousands of tons/year. Although these substances are used in some materials intended for direct ingestion, the main route of exposure is through skin adsorption, being further metabolized and eventually excreted and/or bioaccumulated [123,124]. This dermal absorption may have minor adverse health effects, such as dermatitis, as well as more serious effects, such as mutagenic, carcinogenic, and estrogenic disruptions [125,126]. Accordingly, the presence of residues of many of these components in waters and wastewaters represents a potential hazard for public health and marine organisms. This urges the need to develop methods for simultaneous determination of more than one group of these emerging organic pollutants at the same time.

Several MIP-based analytical techniques are reported in Table 4 for the detection of different types of EDCs. Of the various components of PCPs, endocrine-disrupting chemicals (EDCs) are reported to be the source of many cancerous tumors, birth abnormalities, and other developmental diseases. These compounds are reported to disrupt in the environment via most household and industrial products, which might in turn interact with the hormonal systems of animals and humans [127].

**Figure 8:**
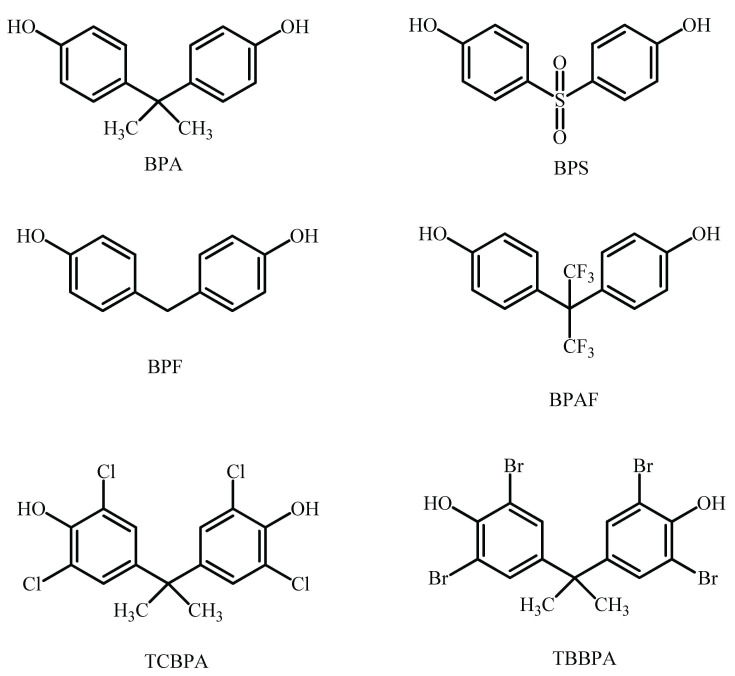
The structure of bisphenols that are most commonly used in polycarbonate plastics.

There are two main groups of environmental carcinogenic EDCs: bisphenols, which can be found in polycarbonate plastics and aluminum can lining, as shown in Figure 8, and phthalates [127]. They can also be found in many pharmaceutical and personal care products available in common beauty stores [123], as well as being used as plasticizers in skincare products containers. It has been shown that bisphenol A (BPA) is related to reduced levels of estradiol and progesterone [128,129,130], while di-2-ethylhexyl phthalate and dibutyl phthalate can result in a decrease in the generation of estradiol and progesterone in both pregnant and cycling rats, suggesting that these chemicals modify steroidogenesis. Thus, bisphenols and phthalates pollutants represent a significant risk to the environment and need to be continuously monitored, especially in water resources where they can be disrupted, as will be discussed briefly and as shown in Table 4.

**Table 4:** Application of MIPs for the determination of selected endocrine disrupting compounds in waters and wastewaters.

Analyte(s)	Monomer(s) Used	Modifiers	Analytical Technique	Tested Sample(s)	Linear Range (µM)	LOD (nM)	Ref.
BPA	MAA	MMIP	UV/λ = 446 nm	Tap, Mineral, and Wastewater	0.44–14.9	131	[131]
BPA	AA	MIP-μPAD	CV, DPV, and fluorescence at λ = 420 nm	Seawater and polycarbonate plastic packaged water	0.004–0.88	2.1	[132]
BPA	MAA/β-CD	Mag-MIPs	UV at λ = 276 nm	Tap water	4.4–1.2 × 10^−3^	---	[133]
BPA	APTES	Fe_3_O_4_@SiO_2_-MIP	fluorescence emission peak at 554 nm (λ_ex_ = 370 nm)	Rainwater, local river, and distilled water	1.0 × 10^−3^–100	0.34	[134]
BPA	APTES	MIP, MWCNTs, CdTe QDs/GCE	CV and DPV	Tap water, river water, and drinking water	0.05 × 10^−3^–0.05	0.015	[135]
BPA	MAA	MIP/Cr_2_O_3_ NPs	Fluorescence, λ_ex_ = 300 nm, and the λ_em_ = 360 nm)	Well water and PC baby bottle	0.04–4.4	15	[136]
BPA	MAA	rGO-Fe_3_O_4_-ZnOMIP/CPE	CV and EIS	Tap water	0.008–15 and 15–95	4	[137]
BPA	MAA	MIP-AuNPs-MCA-rGO/CILE	CV and EIS	Leachates from plastic containers	0.004–18	1	[138]
BPA	[AEIm]PF_6_	CMOF-MIPIL/GCE	CV, EIS, and chronocoulometry	Lake Water and River Water	0.005–5	4	[139]
BPA	4-VP	Mag-MIPs	DPV at GCE	Tap water, milk, and soil	-	133	[140]
BPA	APTES and PheTES	Sol–gel MIP	HPLC-PDA	River water, moat Water, and Bottled water	0.44–4.4	65.7	[141]
BPA	APTES	MIP-nanofibers	UV, λ = 276 nm	Tap water and Wastewater	---	---	[142]
BPA	AAM	MIP nanocomposite composed of RGO, βCD/(GCE)	CV and DPV	Lake water, tap water, and drinking water	0.02–1.0	8	[143]
BPA	MAA	Mag-MIP/AuNPs/CBNPs/SPCE	CV, DPV and EIS	Mineral and tap water	0.07–10	8.8	[144]
BPA	TEOS	SiO_2_@MIP	HPLC-UV	Tap water, Lake water, and Drinking water	-	1.3	[145]
BPA	AAM	MIP–SBSE	HPLC-UV	River water and lake water	4.3 × 10^−5^–0.07	0.01	[146]
BPS	MAA	MIECL	Electrochemiluminescence	Drinking water	2.4 × 10^−3^–50	0.81	[147]
BPS	Pyrrole	MIP-B, N, F-CQDs/AgNPs/GCE	DVP	Mineral water bottles	1 x10^−2^–50	11.2	[148]
TBBPS	APTES	MSPE	HPLC-UV	Tap water, East, and West River	0.018–1.8	0.35	[149]
TBBPA	APTES	WSNs-QDs-MIPs	Fluorescence and HPLC-UV	Pure water	0.025–5	5.4	[150]
DBP	MAA	MIPs@MOF-5	SPE	Tap water	---	---	[151]
DBP, DEP and DMP	MAA	Mag-MIP@MWCNTs	HPLC	Groundwater, Surface water, and Domestic sewage	---	---	[152]
DOP	MAA	MIP/Cu_3_(BTC)_2_@Cu_2_O/ITO(PEC)	Photoelectrochemical	Bottled water	25 × 10^−6^–0.1	0.0092	[153]
DMP, DEP, DBP, DEHP, and DOP	PTMOS and APTES	MGO@mSiO_2_-MIP	GC-MS	River, Lake, Well, and Pond water	---	0.044–0.22	[154]
DBP, BBP, DEP, and DMP	MAA	MIP-SPE	HPLC-MS	Bottle water	0.05–2.6	DBP–3BBP–0.5DEP–1.5DMP–0.88	[155]
DBP	AAM	MQDs–MIPs	Fluorescence	Tap water	5–50	80	[156]
DBP	MAA	MIPs@COOH@SiO_2_	HPLC-UV	Tap water	5.0–30	60	[157]
BP	MAA	MIP-SPME	HPLC- UV	Bottled water, Tap water, and Sea water	8.8 × 10^−3^–0.22	1.1	[158]
MP						1.3	
PP						1.6	
BP	MAA	MIP-coated Silica particles	HPLC- UV	Sunscreen and Swimming pool water	0.048–109.5	14.6	[159]
PP	MAA	MISPE	HPLC- UV	wastewater	0.044–2.77	13	[160]
BA, 4-HBA and SA	4-VP	MMIP	HPLC- UV	Environmental water	0.36–36	BA-634-HBA–7.2SA–144	[161]
BPs	4-VP	MISPE	HPLC-DAD	Tap and river water	1.1–54.9	1.4–4	[162]
BuP	MAA	MIP/MWCNT/GCE	CV, DPV	River water	up to 100	200	[37]
MA, MX, MM, MT, and MK	PTMS and TEOS	MIS	GC–MS	River, sea, and Wastewater	_	MA—9.7 × 10^−3^ MX—9.1 × 10^−3^ MM—2.2 × 10^−3^ MT—6.7 × 10^−3^ MK—5.1 × 10^−3^	[163]
TCS	MMA	MIP nanobeads	Potentiometry	Toothpaste	_	1.9	[164]
TCS	APTES and TEOS	CNTs@TCS-MIPs	HPLC-UV	River water and Lake water	_	_	[165]
BuP	4-VP	MISPE	HPLC-UV	River water	_	_	[38]

**(AA)** acrylamide, **(AAM)** acrylamide, **[AEIm]PF_6_** 1-allyl-3-ethylimidazolium hexafluorophosphate, **(AgNPs)** silver nanoparticles, **(APTES)** 3-aminopropyltriethoxysilane, **(BBP)** butyl benzyl phthalate, **(B, N, F-CQDs)** three-doped carbon quantum dots, **(DBP)** dibutyl phthalate, **(DEHP)** di-(2-ethylhexyl), phthalate **(BA)** benzoic acid, **(BP)** Benzyl paraben, **(BPs)** benzophenones, **(BuP)** butyl paraben, **(β-CD)** β-cyclodextrin, **(CILE)** carbon ionic liquid electrode, **(CMOF)** conductive metal organic framework, **(Cu_3_(BTC)_2_@Cu_2_O/ITO(PEC))** metal organic framework/copper oxide/indium tin oxide/photoelectrochemical sensor, **(DEP)** diethyl phthalate, **(DMP)** dimethyl phthalate, **(DOP)** dioctyl phthalate, **(4-HBA)** 4-hydroxybenzoic acid, **(MK)** Musk ketone(4-tertbutyl-2,6-dimethyl-3,5-dinitro-acetophenone), **(MA)** Musk ambrette(6-tert-butyl-3- methyl-2,4-dinitroanisole), **(MAA)** Methacrylic acid, **(MCA)** mercaptamine, **(MGO@mSiO_2_)** magnetic graphene oxide/mesoporous silica, **(MIECL)** molecularly imprinted electrochemiluminescence, **(MIS)** molecularly imprinted silica, **(MM)** Musk moskene(1,1,3,3,5-pentamethyl-4,6-dinitroindane), **(MP)** methyl paraben, **(MQDs)** magnetic quantum dots, **(MT)** Musk tibetene(1-Tert-butyl-3-methyl-2,4-dinitroanisole), **(MX)** Musk xylene(1-tert-butyl-3,5- dimethyl-2,4,6-trinitrobenzene), **(parabens)** Parahydroxybenzoates, **(μPADs)** microfluidic paper-based analytical devices, **(PheTES)** phenyl triethoxysilan, **(PP)** propyl paraben, **(PTMOS)** phenyl trimethoxysilane, **(SA)** salicylic acid, **(SBSE)** Stir bar sorptive extraction, **(TCS)** triclosan, **(TEOS)** Tetraethoxysilicane, **(SPCE)** Screen Printed Carbon Electrode, **(SPME)** solid phase micro extraction, **(4-VP)** 4-vinylpyridine, **(WSNs)** wrinkled silica nanoparticles.

#### 5.4.1. Bisphenol A

Bisphenol A, being widely used in the production of polycarbonate plastics (≈80%) and epoxy resins (≈18%), is also found in the plastic industry of several products used in everyday life. Reports suggest that BPA is an estrogen-like xenoestrogen affecting the body’s hormonal system [166]. It is known that BPA is carcinogenic even in low quantities, causing abnormal hormone responses and endocrine abnormalities. Approximately 2000 tons of BPA are released into the environment each year due to its widespread use in industrial and household materials. BPA is not degraded or cleaned by wastewater treatment plants; thus, it can be present in natural waters, drinking freshwater sources, riverbeds, air, and diverse food sources. Due to its toxicity, BPA should not be used in baby teethers or plastic bottles. A total of 50 µg kg^−1^ per day is considered safe and tolerated by the United States Food and Drug Administration, but the European Food Safety Authority has established a safe and tolerable dose limit at 0.004 mg kg^−1^ per day. To detect BPA in real-time in the field, it is necessary to create sensing techniques that are speedy, economical, sensitive, selective, easy to prepare samples, and simple to renew on surfaces.

**Figure 9:**
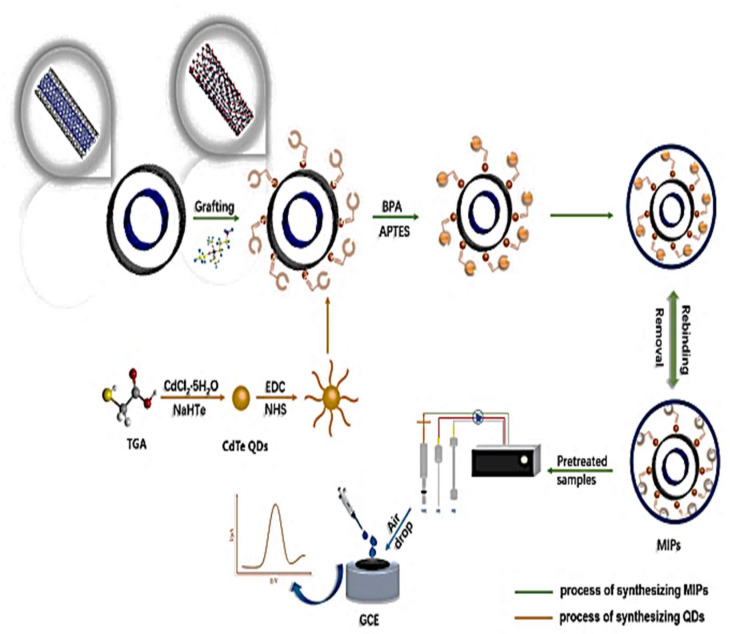
Scheme of the preparation process of BPA electrochemical sensor based on glassy carbon electrode (GCE) and used a mixture of carbon diimide (EDC) and succinimide (NHS) in the preparation of cadmium telluride carboxylate [135]. (Reproduced under permission from Elsevier, doi:10.1016/j.microc.2021.106737).

Recently, BPA was detected using 3-aminopropyltriethoxysilane (APTES) as the functional monomer hydrogen bonded to amine groups of APTES and BPA’s phenolic hydroxyl group on glassy carbon electrode (GCE) in the presence of carboxylated cadmium telluride quantum dots combined with aminated multi-wall carbon nanotubes, as shown in Figure 9 [135]. The sensor showed good selectivity towards BPA and little reactivity to interfering substances within a linear range from 0.05 to 50 mM and a detection limit of 0.015 nM.

#### 5.4.2. Bisphenol S

After the prohibition of BPA use, bisphenol S (BPS) emerged as a key substitute for BPA in consumer products. Bisphenol S, with a much similar chemical structure to that of BPA, shown in Figure 8, contains a sulfone group showing enhanced electron-absorbing abilities and two hydroxyl groups, which makes Bisphenol S more acidic than other bisphenols and more stable than BPA [167]. As an intermediate in the manufacturing of epoxy resins and polycarbonate plastics, bisphenol S can be found in many of our human products today. As a result, individuals are also exposed to BPS in the same way that they are to BPA, though several studies have shown that BPS may be as toxic as BPA in some cases. While BPS had showed similar effects on estrogen and androgen receptor activities as BPA [168], it was found to affect the efficacy on 17α-hydroxyprogesterone of all bisphenol analogues [169].

**Figure 10:**
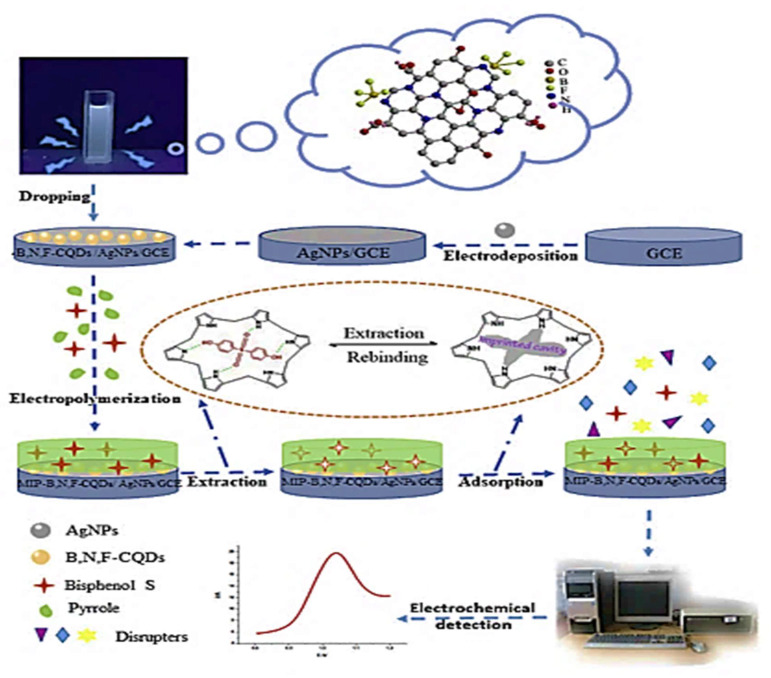
A schematic illustration of the composite of three-doped carbon quantum dots (B, N, F-CQDs) and silver nanoparticles (AgNPs) modified glassy carbon electrode (GCE) for the detection of Bisphenol S [148]. (Reproduced under permission from Elsevier, doi:10.1016/j.aca.2019.03.051).

A BPS electrochemical sensor is reported based on modified GCE using MIP (polypyrrole membrane) as a recognition element and the electron-conducting layer was composed of three-doped carbon quantum dots B, N, and F-CQDs and silver nanoparticles (AgNPs), as shown in Figure 10 [148]. The synergy of B-N and F-CQDs and AgNPs significantly increased sensitivity and resulted in an increase in the electrical signal. In addition, the MIP significantly enhanced the sensor’s selectivity by allowing it to recognize BPS through the imprinted cavities. There was a linear response range of 1 × 10^−8^ to 5 × 10^−5^ M, with a detection limit of 1.12 × 10^−8^ M. This electrode proposed in this article can be considered to satisfy the need for trace-level measurement of BPS in biological and environmental samples.

#### 5.4.3. Brominated Flame Retardants

Brominated flame retardants are also considered as halogenated analogs of Bisphenol A. Tetrabromobisphenol A (TBBPA) is widely utilized in circuit boards and construction materials, while tetrachlorobisphenol A (TCBPA) is used in flame retardant. Both TBBPA and TCBPA can be found in water, soil, sediments, and home dust, as well as in birds, marine life, and humans [170,171]. They were reported to be toxic to the immune and reproduction systems and may also cause endocrine disruption and neurodevelopment [172].

**Figure 11:**
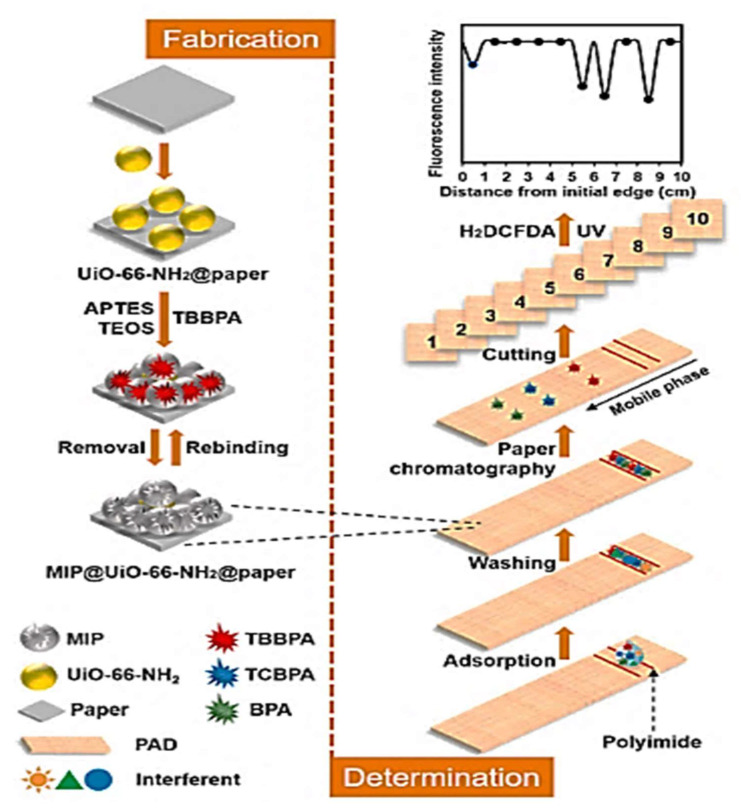
Schematic illustration for the preparation of MIP@ photocatalyst of UiO-66-NH_2_@paper and associated analysis procedures on paper-based analytical device (PAD) for the detection of Tetrabromobisphenol A (TBBPA) and tetrachlorobisphenol A (TCBPA). (Reproduced under permission from Elsevier, doi:10.1016/j.bios.2021.113106).

A paper-based analytical device (PAD) has been developed using molecularly imprinted polymers (MIPs) for TBBPA as a template, and a metal-organic framework of UiO-66-NH_2_, as shown in Figure 11. The adsorption capacity was found to be 120.94 mg g^−1^ with an imprinting factor of 4.07. Due to the PAD’s specific recognition capabilities, TBBPA, TCBPA, and BPA could be effectively separated using paper chromatography with fluorescence detection technique. A photocatalyst, UiO-66-NH_2_, produces reactive oxygen species (ROS) that degrade TCBPA, TBBPA, or BPA in imprinted cavities, and the fluorescence intensity of 2′,7′-dichlorodihydrofluorescein diacetate (H_2_DCFDA), added as an ROS probe, supported the indirect detection of the target substances. For BPA and its halogenated analogues, the detection limits range from 0.14 to 0.30 ng kg^−1^. In total, 91.0 to 105.6% of the analytes were recovered, with RSD values of less than 7.5% [173].

Another recent method for the determination of TBBPA was also reported [150]. Surface imprinted TBBPA polymers on wrinkled silica nanoparticles (WSNs) and CdTe quantum dots (QD) hybrid particles have been used to produce a fluorescent probe for the determination of TBBPA. When coated with MIP, wrinkled silica-QD hybrid particles (WSNs-QDs-MIPs) had a adsorption capacity of 96.5 mg g^−1^ and an imprinting factor of 7.9 towards TBBPA. At optimum conditions, the fluorescence intensity was quenched in the range of 25 to 5000 nM of the TBBPA concentration, at an LOD of 5.4 nM.

#### 5.4.4. Bisphenol F

In another attempt of the industry to respond for the prohibition of BPA, other chemical equivalents, such as bisphenol F (BPF) and bisphenol AF (BPAF), which were thought to be more environmentally friendly replacements, were developed. As with BPA, BPF and BPAF have a similar chemical structure, as shown in Figure 8. There are a variety of industrial uses involving BPF, including lacquers, varnishes, coatings for food packaging, oral prosthetic devices, and dental sealants. With its high-performance monomers, BPAF is utilized as a cross-linker in fluoroelastomer polymers, such as membranes and optical fibers, as well as optical fiber monomers [174]. However, the knowledge on BPA alternatives is limited when it comes to the toxicological characteristics and severe health effects [168]. Based on structural and physicochemical similarities, it is possible that BPA-like compounds might have the same or even greater toxin potential as their analogues. With regards to BPF’s endocrine-disrupting potential, investigations have shown that BPF exhibits estrogenic, antiestrogenic, and antiandrogenic actions equivalent to BPA [175,176,177], while for BPAF, it was reported that it has even stronger estrogenic and anti-androgenic potential than BPA [176,178].

#### 5.4.5. Phthalic Acid Esters

Phthalic acid esters (PAEs), also known as phthalates, are frequently used to communicate and improve the plasticity, flexibility, and durability of the items effectively, as plasticizers. PAEs are included in a variety of goods packaging, consumer products, toys, medical instruments, and construction materials [152]. PAEs can be released easily from plastic materials into the environment because they are not chemically bonded to the polymer matrix [179]. PAEs are suspected of producing human teratogenic and carcinogenic agents, including at a very low level and as a type of synthetic environmental hormone in water environments [151]. PAEs can produce reproductive toxicity for aquatic organisms and can pose a major risk to human health. Some reports showed that this compound could be classified as a pollutant that acts as an endocrine disruptor compound. Di-(2-ethylhexylphthalate) DEHP is reported to be responsible for endometriosis of females as well as for fetus, heart, lungs, kidneys, and liver effects. Dibutyl phthalate (DBP) was reported to be harmful to the central nervous system as well as to embryonic environmental growth, such as changes in the expression and development toxicity of cardiac transcription, pericardial edema, and heart structural abnormalities [180]. In China, DBP and DEHP, for example, were considered to be the mostly detected in water, followed by dimethyl phthalate and diethyl phthalate. In the USA, 147 and 50 ppb, respectively, were indicated as the highest concentrations of DEP and DBP in groundwater.

Recently, a functional corncob biochar (F-CC_3_) was cast on a glassy carbon electrode (GCE) to produce an F-CC_3_ film of functional corncob biochar to increase the effectiveness of the surface area,, then the electrode has been modified by molecularly imprinted polymers (MIPs) synthesized using chitosan as functional monomer, glutaraldehyde as the cross-linker, and DBP as the template compound [181]. The prepared sensor MIP-DBP-CTS/F-CC_3_/GCE showed that the use of F-CC_3_ as inter-layer spacers efficiently inhibited chitosan (CTS) aggregation and resulted in a well-defined porous structure. This modified electrode takes advantages of the molecularly imprinted method and F-CC_3_ biomass materials in order to achieve good selectivity and sensitivity for the detection of DBP by linear relationship in the range of the DBP concentration (0~1.8 μM) with a detection limit of 2.6 nM.

A novel spectroelectrochemical sensor was also presented for the detection of dioctylphthalate (DOP) [153]. Firstly, a thin layer of Cu_2_O was prepared on the surface of indium tin oxide (ITO) by electropolymerization process, then a metal-organic framework (MOF), Cu_3_(BTC)_2_, benzene-1,3,5-tricarboxylate (BTC), was covered on Cu_2_O/ITO to form the heterostructure of Cu_3_(BTC)_2_@Cu_2_O/ITO. This structure showed a strong capacity for adsorption, high stability, and improved photocurrent under visible light radiation. This was then modified with MIP to design MIP/Cu_3_(BTC)_2_@Cu_2_O/ITO by using methacrylic acid as a functional monomer ethylene glycol dimethacrylate (EGDMA) as a crosslinker and 2,2′‒azobisisobutyronitrile (AIBN) as an initiator. At the optimal conditions, this sensor showed a linear range from 25.0 pM to 0.1 μM and an excellent detection limit of 9.15 pM.

### 5.5. Pharmaceutical Products

Pharmaceutical drugs and their residues have been found in surface waters, raising concerns about their potential adverse effect on human health if inadvertently ingested through contaminated food and water sources. Pharmaceuticals have predominantly entered ground and surface water through wastewater treatment plant (WWTP) effluents due to their extensive use. Pharmaceutical medications are water soluble and have a high polarity, allowing them to easily evade wastewater treatment facilities. Others undergo incomplete breakdown, and certain degradation products can even be de-conjugated back to their biologically active forms. Table 5 represents examples for the application of MIPs in the determination of pharmaceutical residues in waters and wastewaters.

Despite the fact that medications are present in low concentrations in the aquatic environment (μg-ng L^−1^), their residues can produce ecotoxic effects, hormone disturbance, and drug resistance [182]. There are different types of pharmaceuticals that are eligible to be found in wastewater, such as antibiotics, antidepressant, antiretroviral, analgesics, anticonvulsant, antiseptic, non-steroidal inflammatory drugs, and hormones [182,183]. Antibiotics are shown to present a new class of environmental contaminants that have the potential to damage human and animal health as well as ecosystem survival. The development of bacterial resistance to existing antibiotics as a result of continuous exposure is largely responsible for the rise in awareness of antibiotic pollution in the environment [184].

A sensor based on molecularly imprinted polymer was developed for erythromycin (Ery) in water samples. Poly(m-phenylene diamine) was electropolymerized on gold screen printed electrode at 0.63 V. The different steps of the MIP preparation were characterized using electrochemical techniques, such as cyclic voltammetry (CV) and electrochemical impedance spectroscopy (EIS). Ery-MIP/SPE sensor demonstrates appreciable affinity for Ery and low analytical limits, LOD = 0.1 nM, LOQ = 0.4 nM [184].

Antidepressants, also known as selective serotonin reuptake inhibitors (SSRIs), are one of the commonly found pharmaceuticals in the environment. MIPs were applied for extraction of SSRIs in water using methacrylic acid as monomer, ethylene glycol dimethacrylate (EGDMA) as cross linker, and 2,2′-azobis(2,4-dimethyl valeronitrile) as initiator. The maximal capacity of MIP for sertraline in water is 72.6 mg g^−1^, and the highest imprinting factor is 3.7. High performance liquid chromatograph coupled with a diode array detector (HPLC-DAD) was used for the different detections. Despite the small surface area of the synthesized polymers that ranged between 27.4 and 193.8 m^2^ g^−1^, as compared to that of the activated carbon at 1400 m^2^g^−1^, their sorption capabilities in wastewaters were generally superior [185].

Carbamazepine (CBZ), an antiepileptic and mood-stabilizing medicine used mostly to treat epilepsy and bipolar disorder, known for its toxicity, is a developing contaminant in water. MAA-based porous molecularly imprinted polymer was applied for solid phase extraction for selective and preconcentration of carbamazepine (CBZ) from various water samples. This approach was shown to be extremely suited for the quantification of CBZ, with good recovery (87.2–99.4%), low detection limit (82 pg mL^−1^), and reusability of adsorbent [186].

Molecularly imprinted polymers are capable of excellently binding to different classes of pharmaceutical compounds, regardless of their structure or pharmacological activity, and thus, they have high potential to be applied in wastewater treatment planets, unlike traditional adsorption materials, such as clays and carbonaceous adsorbents, which depend mainly on surface or physical adsorption. Additionally, the use of MIPs for pharmaceutical removal is more cost-effective compared to ozonolysis, photochemical, or electro-decomposition methods and is much safe compared to the use of biological degradation, which might lead to the production of some toxic side products that can be considered hazardous materials.

**Table 5:** Application of MIPs in the determination of pharmaceutical residues in waters and wastewaters.

Kind of Pharmaceutical	Analyte	Monomer	Modifiers	Analytical Technique	Sample	Linear Range	LOD	Ref.
Antibiotics	Ciprofloxacin	MAA	Ch-AuMIP	CV and DPV	Mineral and tap water	1–100 μmol L^−1^	210 nmol L^−1^	[187]
	Nalidixic acid	MAA	NA-MIP	UV–Vis spectrophotometry	Pills and seawater	1–150 μg L^−1^	0.2 μg L^−1^	[188]
	Erythromycin	mPD	Ery-MIP	DPV	Tap water	2–16 nM	0.1 nM	[184]
	Norfloxacin	MAA	MIP-CP	FL spectrophotometry	Aqueous solutions	10–100 ng mL^−1^	2.59 ng mL^−1^	[189]
	Chloramphenicol	MAA	MIPs-BiOBr-ITO	EIS	Water sample	10^−2^–10^−3^ ng mL^−1^	3.02 pg mL^−1^	[190]
Antidepressant	Venlafaxine	p-vinyl benzoic acid	MASE-MIP	HPLC	Water samples	5–100 ng mL^−1^	0.13 ng mL^−1^	[183]
	Sertraline	MAA	SER-MIPs	HPLC-DAD	Wastewater	2.79–120.7 μg L^−1^	0.41 μg L^−1^	[185]
Antiretroviral	Efavirenz	2-VP	MIPs	HPLC-DAD	Wastewater	-	-	[191]
Analgesics	ketoprofen	2-VP	MIPS	HPLC	Wastewater	1–1000 μg L^−1^	0.3 μg L^−1^	[192]
	Naproxen	MAA	MIP-MWCNTs-EME	HPLC-UV	Wastewater	1–100 μg L^−1^	0.3 μg L^−1^	[193]
	Acetaminophen	MAA	Ace-MIPs	UV–Vis spectrophotometry	Pills and seawater	1–300 nmol L^−1^	0.34 nmol L^−1^	[188]
	Acetaminophen	APTES	MIP-capped L-cys-CdSe/ZnS QD	Fluorescence emission spectrophotometry	Water sample	-	-	[194]
Anticonvulsant	Carbamazepine	MAA	PMIP-CBZ	HPLC	Various water samples	0.2–250 ng mL^−1^	0.082 ng mL^−1^	[186]
	Phenytoin	AAm	MISPE	HPLC	Wastewater	2–30 μg mL^−1^	0.7 μg mL^−1^	[195]
Antisepatic	Triclosan	AAm	Au-SPE-MIP	DPV and EIS	Wastewater	0.1–1000 pg mL^−1^	o.23 pg mL^−1^	[196]
Non-steroidal anti- inflammatory drug	Fenoprofen	2-VP	MIPs	HPLC	Wastewater	24–58 ng mL^−1^	0.64 ng mL^−1^	[197]
	Diclofenac	AT	MIP-DCF	UV–Vis spectrophotometry	Water sample	1–25 mg L^−1^	-	[198]
	Meloxicam	MAA	ZnO-NPs@MEL-MIP The	UV–Vis spectrophotometry	Water media	0.1–10 mg L^−1^	0.008 mg L^−1^	[199]
Hormones	Progesterone	PY	MIPs	GC-FID	Hospital wastewater and tap water	1.25–5000 ng mL^−1^	0.625 ng mL^−1^	[200]
	Estriol	MAA	MMIP	HPLC	Water sample	-	-	[201]
	Estrone	MAA	MMIP	HPLC	Water sample	-	-	[201]

**(AT)** allylthiourea, **(EIS)** electrochemical impedance spectroscopy, **(FTIR)** Fourier transmission infrared spectra, **(GCE)** glassy carbon electrode, **(GC-FID)** Gas chromatography coupled with Flame Ionization Detector, **(^1^H NMR)** Nuclear Magnetic Resonance, **(HPLC-DAD)** high performance liquid chromatograph coupled with a diode array detector, **(MAA)** Methacrylic acid, **(mPD)** m-phenylenediamine, **(PY)** pyrrole, **(UV)** ultraviolet, **(2-VP)** 2-vinylpyridine.

## 6. Conclusions

Based on the data discussed in this review, it seems to be obvious that MIPs are presenting a promising tool not only for detecting the existence of different types of contaminants, but also show possibility for their removal and wastewater treatment. The adsorption properties and sensitivity were superior to widely used other extraction and detection methods. Being of low cost and ease of preparation enhances their applicability to real applications. The design of the MIPs can be tailored according to the properties of the target template and its hydro-interactive nature. Currently, there are several manufacturers that started to offer commercially different types of solid phase extraction MIPs cartridges for many environmental pollutants, which shows that the potential future of MIPs as excellent sorbents for clean water is getting close to reality.

## Figures and Tables

**Table 3:** Application of MIPs based on solid phase extraction and sensors for the detection of pesticides in water samples.

Pesticide	Analyte	Functional Monomer	Polymerization Method	Modifier	Analytical Technique(s)	Sample(s)	Linear Range	Limit of Detection	Ref.
Fungicides	Dicloran	MAA	Bulk	-	SWV	River and Tap water	1 × 10^−6^ to 1× 10^−9^ mol L^−1^	4.8 × 10^−10^ mol L^−1^	[87]
	Tributyltin	APTES	Sol-gel	Fe_3_O_4_ MNPs	EIS	Sea water	5.0 × 10^−3^–5.0 × 10^3^ nM	5.4 × 10^− 3^ nM	[88]
Herbicides	Bensulfuron-methyl	MAA	Imprinted magnetic microspheres	-	HPLC	Water	0.04−0.8 µM	6.4−9.5 nM	[89]
	Butachlor	AGG/4-VP	Precipitation	-	HPLC	Water	0.1–200 µg L^−1^	0.03–0.06 µg L^−1^	[90]
	Chloridazon	2-VP and MAA	Bulk	MWCNTs	DPV	Ground, surface, seawater, and drinking water	5.0 × 10^2^–4.0 × 10^5^ nM	62 nM	[91]
	4-Chlorophenol	o-PD	Electro-polymerization	ZnO NPs	SWV	Wastewater	2.0 × 10^2^–1.7 × 10^5^ nM	40 nM	[92]
	2,4-dichlorophenol	MAA	Bulk	Microgel suspension, Ch, Nafion	DPV	Tap, river, and drinking water	5.0 × 10^3^–1.0 × 10^5^ nM	1.6 × 10^3^ nM	[93]
		MAA	Bulk	GO	DPV	Lake water	4–1.0 × 10^4^ nM	0.5 nM	[94]
		EDOT	Electro-polymerization	-	DPV	Lake, river, and tap water	0.21–3.0 × 10^2^ nM	0.07 nM	[95]
	2,4-dichlorophenoxyacetic acid	MAA	Precipitation	-	HPLC-UV	Sea, river, and tap water	-	1.25 and 1.80 µg L^−1^	[96]
	Diuron	MAA	Bulk	MWCNTs	SWV	River water	52–1.3 × 10^3^ nM	9.0 nM	[97]
	Glyphosate	Py	Electro-polymerization	-	DPV	Tap water	30–4.7 × 10^3^ nM	1.6 nM	[98]
	Hexazinone	2-VP	Bulk	-	DPV	River water	0.019–0.11 nM	2.6 × 10^− 3^ nM	[99]
	Propazine	MAA	MIP-HFs	-	HPLC-DAD	Tap and river water	0.1–25 µg L^−1^	0.03–0.1 µg L^−1^	[100]
		MAA	MIP-SPME	-	HPLC-DAD	Tap water	-	0.022–0.030 µg L^−1^	[101]
	Paraquat	Py	Electro-polymerization	EBB	DPV	Dam water	5.0 × 10^3^ –5.0 × 10^4^ nM	2.2 × 10^2^ nM	[102]
	Thifensulfuron- methyl	Acrylamide	Imprinted electrochemiluminescence	-	ECL analyzer	Water	5.0 × 10^−10^–1.0 × 10^−7^ M	0.32 nM	[103]
Insecticides	Chlorpyrifos	MAA	Bulk	-	DPV	River water	0.1–1.0 × 10^4^ nM	4.1 nM	[104]
		Py	Electro-polymerization	C_3_N_4_ NTs, GQDs	SWV	Industrial wastewater	1.0 × 10^−2^–1 nM	2.0 × 10^− 3^ nM	[105]
		PATP	Electro-polymerization	-	GC-MS	Water	0.01–100 ng mL^−1^	7.4 pg mL^−1^	[106]
	Cypermethrin	Ph	Electro-polymerization	Fe@AuNPs, 2D-hBN	DPV	Waste water	1.0 × 10^−3^–10 nM	3.0 × 10^−5^ nM	[107]
		DA and RC	Electro-polymerization	Ag–N@ZnO, CHAC	EIS	Tap water and soil	2.0 × 10^−4^–8 nM	6.7 × 10^− 5^ nM	[108]
	Diazinon	MAA	Bulk	-	SWV	Well Water	2.5–1.0 × 10^2^ nM1.0 × 10^2^–2.0 × 10^3^ nM	0.79 nM	[109]
		MAA	Bulk	MWNTs	SWV	Tap and river water	0.5–1.0 × 10^3^ nM	0.13 nM	[110]
	4,4ˈ-dichlorobenzhydrol	1-allyl-3- ethylimidazolium hexafluorophosphate/MAA	Suspension	-	GC	Water	1.0–100 ng mL^−1^	0.12–0.26 ng mL^−1^	[111]
	Methyl-parathion	Ph	Electro-polymerization	N-GS	CV	River water	3.8 × 10^2^–3.8 × 10^4^ nM	38 nM	[112]
	Parathion	MAA	Bulk	-	SWV	Tap, river, and lake water	1.7–9.0 × 10^2^ nM	0.5 nM	[113]
	Phosalone	APTES	Sol-gel	Pt-UiO-66	SWV	Lake water and soil	0.50–2.0 × 10^4^ nM	0.078 nM	[114]
	Triazophos	PATP	Electro-polymerization	Luminal	ECL	Tap water, reservoir water, and river water	0.1–1.0 × 10^3^ nM	0.058 nM	[115]

**(AGG)** alkenyl glycosides glucose, (**APTES)** (3-Aminopropyl)triethoxysilane, (**AuNPs)** gold nanoparticles, **(CFP)** carbon fiber paper, **(CHAC)** activated carbon prepared from coconut husk, (**C_3_N_4_ NTs)** carbon nitride nanotubes, **(CNTs)** carbon nanotubes, **(CPE)** carbon paste electrode, **(CPME)** carbon paste microelectrode, **(CV)** cyclic voltammetry, **(DA)** dopamine, (**2D hBN)** two dimensional hexagonal boron nitride, **(DPASV)** differential pulse anodic stripping voltammetry, **(DPV)** differential pulse voltammetry, **(EBB)** eriochrome blue-black B, **(ECL)** electrochemiluminescence, **(EIS)** electrochemical impedance spectroscopic, **(EDOT)** 3,4-ethylenedioxythiophene, **(GCE)** glassy carbon electrode, **(GO)** graphene oxide, **(GQDs)** graphene quantum dots, **(2-HEMA)** 2-hydroxyethyl methacrylate, **(LSSV)** linear stripping sweep voltammetry, **(LSV)** linear sweep voltammetry, **(MAA)** methacrylic acid, **(MAC)** N-methacryloyl-L-cysteine, **(MWCNTs)** multiwalled carbon nanotubes, **(N-G_S_)** nitrogen doped graphene sheet, **(o-PD)** o-phenylenediamine, **(PATP)** p-aminothiophenol, **(PGE)** pencil graphite electrode, **(Ph)** phenol, **(PtNPs)** platinum nanoparticles, **(Pt-UiO-66)** Zr-based metal–organic framework catalyst, **(Py)** pyrrole, **(QDs)** quantum dots, **(RC)** resorcinol, **(SPAuE)** screen printed gold electrode, **(SPCE)** screen printed carbon electrode, **(SWV)** square wave voltammetry, **(ZnO NPs)** zinc oxide nanoparticles, **(2-VP)** 2-vinylpyridine.

## Data Availability

Not Applicable.
